# Enzyme therapy as a promising strategy to overcome multi-drug resistance in pathogens: current advances, key challenges, and future directions

**DOI:** 10.1039/d5ra06932g

**Published:** 2026-01-26

**Authors:** Saloni Saini, Rahul Yadav, Ranjana Das, Chandan Singh

**Affiliations:** a Department of Biochemistry, Central University of Haryana Mahendergarh 123031 India; b Department of Biochemistry, Institute of Science, Banaras Hindu University Varanasi 221005 India chandansingh@bhu.ac.in +917379180379; c Department of Pharmaceutical Engineering & Technology, Indian Institute of Technology, BHU Varanasi 221005 India ranjanadas@bhu.ac.in +918840292279

## Abstract

Multi-drug resistance (MDR) poses a serious threat to global health due to increasing death rates, as antibiotics are rendered ineffective, resulting in fatal infections as an outcome of treatment failures, which make them cost-ineffective in the healthcare sector. The excessive use of antibiotics in therapies for human diseases, along with their usage in farm animals and fishes, causes selective pathogens to develop resistance against multiple drugs. In the era of growing drug resistance, enzyme therapy is emerging as a valuable alternative. Enzymes such as endolysins, DNase I, dispersin B, nanozymes, lysostaphin and alginate lyases are involved in combating multidrug-resistant pathogens. These enzymes can degrade the peptidoglycan layer, disrupt biofilms and neutralize resistance factors. Although current advantages such as phage-derived lysins, biofilm-degrading enzymes, enzyme-antibiotic synergy, targeted delivery systems and engineered enzymes are explored, challenges such as low stability, unclear delivery mechanisms, and large-scale production of enzymes remain, which are addressed in the present review along with future directions. Continuous research into enzyme engineering, nanocarrier systems, and synergistic combination therapies could provide effective, sustainable solutions to invade MDR pathogens. The present review highlights the potential of enzyme therapy in overcoming resistance by categorizing these enzymes according to their mode of action, target site, and therapeutic function. These insights reveal that enzyme therapy is a promising strategy to fight against drug-resistant pathogens.

## Abstract 2

Enzyme-based therapies for multi-drug resistant infections.

## Graphical abstract

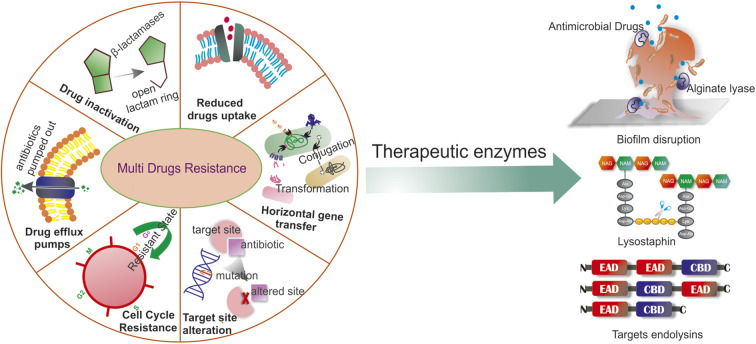

## 1 Introduction

**Fig. 1:**
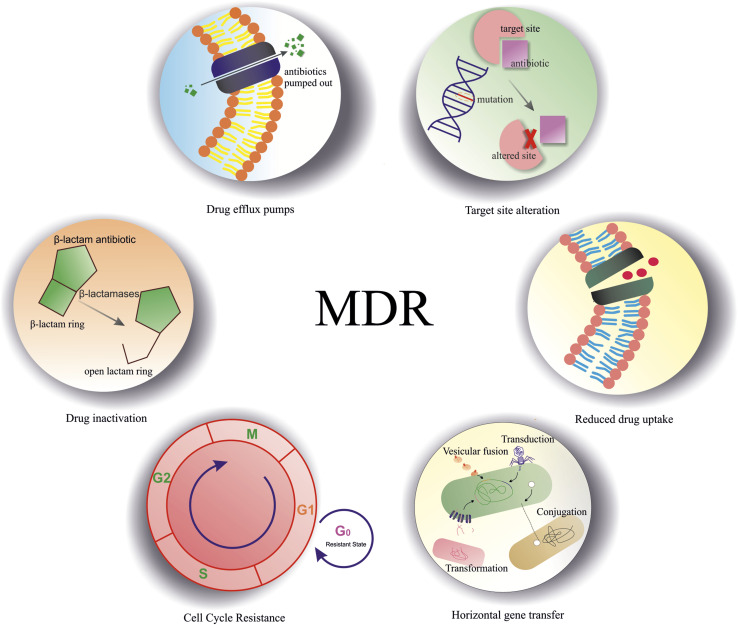
Illustration of the major mechanisms contributing to multidrug resistance (MDR). These include drug efflux pumps, which actively transport antibiotics out of the cell, lowering intracellular drug concentrations; target site alteration, which involves modifications in the bacterial components; and reduced drug uptake, where decreased membrane permeability or loss of porin channels limits antibiotic entry. Horizontal gene transfer enables the acquisition of resistance genes allowing rapid dissemination of resistance traits. Cell cycle-related resistance refers to altered susceptibility during dormancy states. Lastly, drug inactivation occurs through enzymatic degradation or chemical modification of the drug molecule, rendering the antibiotic ineffective.

The ability of a drug molecule to lose its efficacy due to certain mutations, decreased drug permeability, single-nucleotide polymorphisms (SNPs), or enzymatic transformations like hydrolysis, group transfer, and oxidation–reduction reactions is known as drug resistance, and it is currently the most worrisome health risk.^1^ Multi-drug resistance (MDR) arises when a particular strain develops resistance against different antibiotics. Drug-resistant microbes pose a serious health risk since new drugs are unavailable to treat these infections, and MDR is mainly caused by the overuse of broad-spectrum antibiotics such as penicillin, azithromycin, and carbapenems.^2–5^ High prevalence of drug resistance reduces the effectiveness of drugs and narrows the treatment options for patients with tumors and infectious diseases.^6–9^ Pathogenic evolution drives resistance development against essential antimicrobials, including antibiotics, antivirals, antifungals and antiprotozoals. Past research on drug resistance focused on the cellular mechanism underlying the process. The other factors responsible for MDR are low diffusion of drugs into the target molecule, inactivation of drugs due to their destruction or transfer of any functional group like adenyl, acetyl and phosphoryl groups, active drug efflux or any modification in the target (where the drugs interact), cell cycle-related resistance and horizontal gene transfer (Fig. 1).^10^ Various compounds are reported to avoid resistance development, which include antimicrobial peptides (AMPs), and studies confirmed that AMP DP-7 avoids biofilm formation by *Pseudomonas aeruginosa* isolates and prevents cell wall formation by inhibiting the expression of genes associated with lipopolysaccharide (LPS) synthesis in Gram-negative bacteria.^11^ Infections can be categorized as nosocomial (hospital-acquired) and community-acquired. If the symptoms of the early infection phase occur within 48 hours of hospitalization, then it must be a community infection, but if they occur after 48 hours, then it is considered a nosocomial infection.^12^ Resistance against any medication can be assessed through the minimum inhibitory concentration (MIC) score, which is the minimum concentration of any antibiotic capable of inhibiting the growth of a particular pathogen.^13,14^

On the work front, there are numerous ongoing research projects to discover new drug molecules that will assist in treatment against drug-resistant pathogens. According to a recent report, the antibiotic resistance could be as old as 2 billion years and as young as 40 million years, as suggested by paleogenetic studies and genome sequencing, and it is still happening on earth over time.^15^ Long ago, “Father of Antibiotics”, Alexander Fleming claimed that if a lower dose of penicillin is used for a long time, then there might be a higher chance of resistance development.^16^ There exist several antimicrobial agents that are classified based on their mechanism of action: some inhibit protein synthesis, some inhibit nucleic acid synthesis, some interfere with the metabolic pathways, some prevent cell wall formation, and many more.^17^ If a particular bacterium becomes resistant to a specific antibiotic drug, then its upcoming generation will gain resistance on its own, until and unless any mutagenesis occurs. Sometimes, bacteria gain persistence towards antibiotics, which means they are in a dormant state for a short period.^18^

The major problem while dealing with drug resistance is biofilm formation. Biofilms are clusters of microorganisms that are embedded on a surface and made up of an extracellular polymer matrix that contains polysaccharides, nucleic acid and some proteins.^19^ The species that efficiently form biofilms are *Staphylococcus aureus* and *Pseudomonas aeruginosa*, which are difficult to treat with conventional antibiotics and can cause fatal diseases in the infected patients.^20^ The biofilm-associated pathogens are difficult to treat as they are embedded inside the surface matrix, which traps the drug molecule within itself, ultimately rendering it ineffective.^21^ In the food industry, the inclusion of nanoparticles in bio-films is a new approach to enhance the shelf-life of packaged food by reducing the chances of contamination of food by these pathogens.^22^ One of the worst infections, ESKAPE, is a serious worry when dealing with drug-resistant microorganisms since they can develop resistance to several antibiotic compounds. ESKAPE indicates different species in the order: *Enterococcus faecium*, *Staphylococcus aureus*, *Klebsiella pneumoniae*, *Acinetobacter baumannii*, *Pseudomonas aeruginosa*, and species of the genus *Enterobacter*.^23^

A variety of enzymes play a crucial role in enhancing the resistance mechanism of pathogenic microorganisms. One example is β-lactamase, an enzyme that contributes to resistance in Gram-negative bacteria. β-Lactamases are the enzymes involved in the degradation of β-lactam antibiotics by hydrolysing the β-lactam amide bond, which in turn prevents them from interacting with penicillin-binding proteins (PBPs) and causing the disruption of bacterial cell wall synthesis.^24,25^ The pathogen *Staphylococcus aureus* develops resistance by enzymatic mechanism.^26^ Thus far, almost 1000 β-lactamases have been discovered, and many more are yet to come.^27^ Another example are chloramphenicol acetyltransferases (CATs), which chemically modify the chloramphenicol antibiotic and interfere with the protein synthesis process by hindering peptidyl transfer on the 50S ribosome subunit.^28,29^ Further investigation can be carried out to discover new antibiotic therapies against drug-resistant pathogens by isolating the microorganism from several fungal species.

Enzyme therapies such as endolysins, dispersin B, and DNases offer targeted antibiofilm activity and reduced risk of resistance, making them attractive candidates for treating MDR infections. However, small-molecule drug discovery targeting novel bacterial mechanisms continues to play a pivotal role in the fight against resistant pathogens. Membrane-active small molecules have demonstrated broad-spectrum antibacterial activities, the ability to eradicate biofilms, and a low propensity for resistance growth. The antibacterial pipeline underscores that innovative small-molecule scaffolds and targets remain vital for addressing unmet clinical needs and complementing alternative modalities such as enzyme therapies. These complementary approaches highlight the importance of a multifaceted strategy in addressing multidrug resistance. Classical screening of microorganisms from rich ecological environments continues to be fruitful, and recent strategies have expanded into highly diverse or previously inaccessible habitats such as marine organisms, algae, insects, and symbiotic microbiota. These searches have uncovered novel compounds like lugdunin, a macrocyclic thiazolidine peptide from *Staphylococcus lugdunensis*, which demonstrates strong action against Gram-positive pathogens including *Staphylococcus aureus*. Parallel work using metagenomic mining and bioinformatic prediction of biosynthetic gene clusters has yielded additional promising molecules such as tetarimycin—a tetracyclic antibiotic active against MRSA from soil microbiomes—and humimycin, which inhibits lipid II flippase and synergizes with β-lactam antibiotics.^30,31^

To overcome the enzyme-mediated resistance, novel strategies must be developed. In order to counter, an enzyme-based therapy has been investigated that targets the mechanism involved in resistance enhancement and involves both the enzyme and inhibitors. This approach could help fight against drug resistance. Enzymes are biocatalysts with high catalytic efficiency to target specific sites in a particular reaction.^32–38^ The very first enzyme approved by the Food and Drug Administration (FDA) back in the 1980s as a therapeutic approach was Alteplase, which is used for treating acute ischemic stroke patients. The present review focuses on various approaches based on enzymes as an emerging therapeutic tool for combating multidrug resistance. The enzymes included in Table 1 were selected based on the literature from experimental or clinical studies reported and categorized for an improved understanding of their therapeutic relevance.

**Table 1:** Classification of enzymes based on their EC number, target, source, and size

Enzymes	EC number	Source	Target site	Size (kDa)	Ref.
Endolysins	EC 3.2.1.17	Bacteriophages	Degrades the peptidoglycan layer	25	39
DNase I	EC 3.1.21.1	Bovine pancreas	Breaks 3′,5′-phosphodiester bond of extracellular DNA	30–45	40
Dispersin B	EC 3.2.1.52	*Aggregatibacter actinomycetemcomitans*	Hydrolyzes PNAG, a component of biofilms	40	41
Nanozyme (POD)	EC 1.11.1.7	Various inorganic materials	Generate ROS to target the bacterial cell membrane	40–50	42
Lysostaphin	EC 3.4.24.75	*Staphylococcus simulans*	Cleaves pentaglycine peptide present between NAM molecules	27	43
Alginate lyase	EC 4.2.2.3, EC 4.2.2.11, EC 4.2.2	*Flammeovirga* sp., *Serratia marcescens*, *Saccharina japonica*	Disrupts biofilms by cleaving 1–4 glycosidic bond	30–35	44
Lactonase	EC 3.1.1.81	*Stenotrophomonas maltophilia*	Inhibits quorum sensing molecules	30–40	45

## 2 Exploring diverse enzyme-based therapies for combating MDR pathogens

### 2.1 Endolysins

Endolysins (3.2.1.17) are bacteriophage-encoded antibacterial enzymes that selectively degrade bacterial peptidoglycan, leading to cell lysis.^23^ They are particularly effective against Gram-positive bacteria as they have their cell wall in an exposed environment.^46^ Additionally, they can degrade bacterial biofilms and can be used in biosensors for rapid pathogen detection.^47,48^ It was reported that the endolysin phi11 shows potent antimicrobial activities against various *Staphylococcus* strains including *Staphylococcus aureus*, *Staphylococcus epidermidis*, *Staphylococcus hyicus*, *Staphylococcus simulans*, *Staphylococcus xylosus* and *Staphylococcus wraneri*.^49^ The endolysins LysAB54, LysP53 and LysPA26 showed antibacterial activities against MDR Gram-negative bacteria such as *Acinetobacter baumannii*, *Pseudomonas aeruginosa*, *Klebsiella pneumoniae*, and *Escherichia coli.*^50^ The reported that endolysins Abtn-4 and LysSS have a wide range of bactericidal activities against a wide range of Gram-positive and Gram-negative bacteria including *Escherichia coli*, *Acinetobacter baumanii*, *Pseudomonas aeruginosa*, *Klebsiella pneumoniae*, *Salmonella* spp. and *Staphylococcus aureus*.^51,52^

**Fig. 2:**
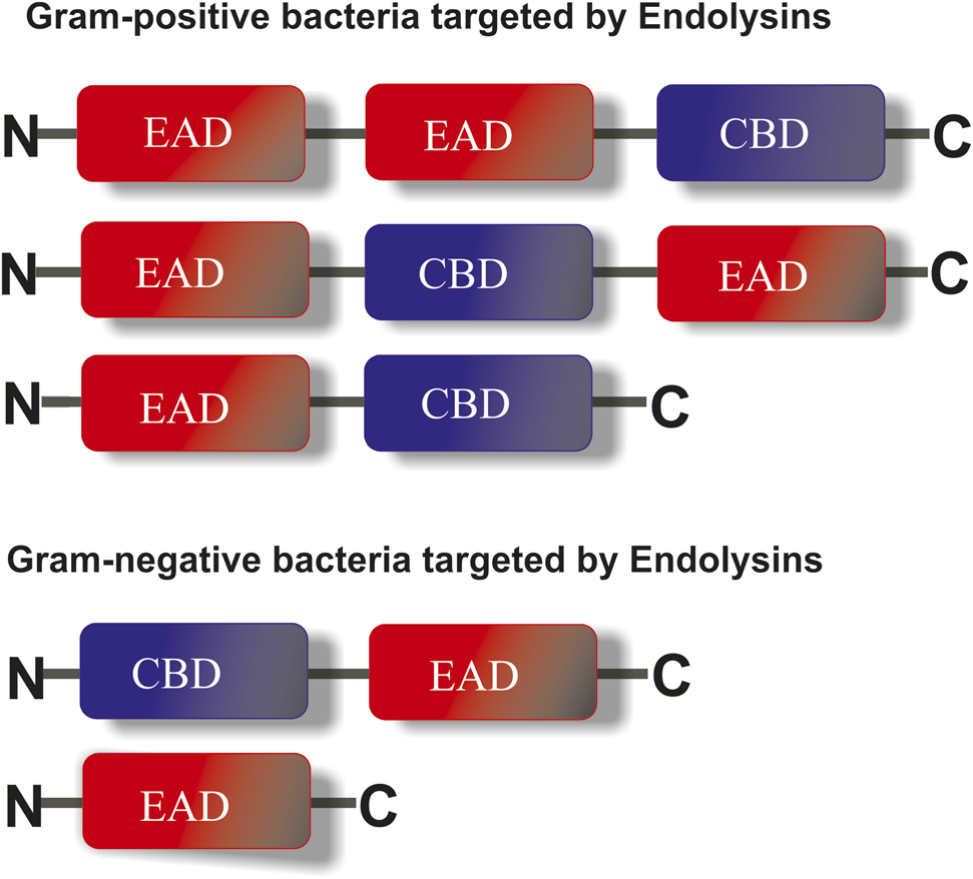
Domain arrangements of endolysin in Gram-positive and Gram-negative bacteria. Gram-positive bacteria have at least one CBD with one or two EAD domains, but Gram-negative bacteria do not have any CBD due to variation in their cell wall organization.

Endolysins are a 25 kDa monomeric protein having two functioning domains: a cell wall-binding domain (CBD), to regulate the recognition of endolysins and bind them to the bacterial cell, and an enzyme active domain (EAD) that catalyses the cell wall hydrolysis.^53,54^ Once the peptidoglycan layer is disorganised, the production of endolysins occurs. As depicted in Fig. 2, the different endolysin domains for Gram-positive and Gram-negative bacteria are differentiated based on their different cell wall composition. In Gram-positive bacteria, there is at least one CBD domain with one or more EAD domains, while in Gram-negative bacteria, CBD domains are comparatively few. The two lysis mechanisms, “lysis-from-without” and “lysis-from-within”, work in the disruption of Gram-positive and Gram-negative bacteria.^55^ Gram-positive bacteria have a thick peptidoglycan layer, so the “lysis from-without” mechanism works in which endolysins lyse the bacterial cell externally without the need to get their viral RNA replicated inside the host pathogens; however, Gram-negative bacteria get destroyed by the “lysis-from-within” mechanism. The different CBDs reported are choline, Cpl-7, lysin motif, and bacterial Src homology 3.^39^

In Gram-negative microorganisms, peptidoglycan is degraded through two distinct mechanisms: (1) the holin–endolysin system and (2) the pinholin-SAR endolysin pathway.^49^ In the initial phase, holin, which are hydrophobic proteins, form small pores in the host's inner membrane at the cytoplasmic side, facilitating the release of endolysin into the periplasm to degrade peptidoglycan. Lysis is subsequently triggered in the final stage by pinholin-induced membrane depolarization, activating the SAR endolysin.^56^

Typically, phages use a two-part spanin complex consisting of *o*-spanin, present at the outer lipoprotein membrane, and *i*-spanin, present internally with a coiled-coil periplasmic domain.^57^ The term “spanins” comes from their ability to bridge both bacterial membranes. However, some phages utilize a single-molecule spanin that extends to the periplasm, containing a C-terminal transmembrane domain and an N-terminal lipoprotein signal.^58^ The peptidoglycan meshwork restricts spanin activity, aligning its function with the prior lysis steps of holin and endolysin. These proteins ultimately induce the lysis of bacteria.

Endolysins have a wide range of applications, like Cpl-1 endolysins used to treat infections caused by different serotypes of *Streptococcus pneumoniae*, and if Cpl-1 is present in a dimer form, its efficacy will increase two times.^39^ Moreover, the PlyC and PlyPy lysins are effective against *Streptococcus pyogenes* biofilms. Another endolysin, which is a fusion product of Cpl-7 and Cpl-1 to form Cpl-711 lysin, resists the adhesion of *Streptococcus pneumoniae* to the host membrane.^55^ LySMP endolysin has been employed to combat biofilm formation by *Streptococcus suis*.^54^

#### 2.1.1 Structure and mechanism of action of endolysins

The holin–endolysin system is widely considered as the central mechanism by which phages orchestrate host cell lysis, but accumulating evidence reveals the existence of holin-independent lysis pathways too. Coliphages P1 and 21 express the endolysins LyzP1 and R21, which feature a signal-arrest-release domain at the N-terminus, which recruits them into the secretory (Sec) translocon, allowing secretion without holin-mediated membrane disruption.^59,60^ The endolysin of phage AP1 contains a C-terminal membrane-insertion motif, enabling its movement across the membrane through the Sec system. In the swi2 phage, although a holin gene is present, the two encoded endolysins themselves carry out lysis both inside and outside the cell, while the holin lacks membrane-penetrating activity. Bacteriophage endolysins exert their lytic activity by cleaving defined glycosidic bonds within the bacterial peptidoglycan.^61^ The PG layer is made of a repeating polysaccharide consisting of *N*-acetylglucosamine and *N*-acetylmuramic acid joined by β-1,4 bonds.^62^ Attached to the muramic acid residues are tetrapeptide stems that crosslink with neighbouring strands, generating a three-dimensional, mesh-like lattice. This structural polymer is a fundamental component of the bacterial cell wall, enabling cells to withstand high internal osmotic pressure and retain their shape. While the carbohydrate backbone is conserved, the peptide side chains and their linkages differ among bacterial species. Endolysins fall into five categories: muramidases, glucosaminidases, lytic transglycosylases, amidases, and endopeptidases. Muramidases, glucosaminidases, and lytic transglycosylases all target the glycan component of PG by cleaving the MurNAc–GlcNAc linkage.^63^ Even though muramidases and lytic transglycosylases target the same bond, muramidases use water to hydrolyse it, whereas lytic transglycosylases cut the bond through a non-hydrolytic mechanism.

Each Gram-positive bacterium presents a characteristic set of secondary cell wall polymers (SCWPs), embedded within the lipid bilayer–PG matrix, shaping both its surface appearance and electrostatic profile. Endolysins rely on their CBD to selectively recognize these SCWPs, allowing precise docking before the EAD degrades the PG. Once the CBD is removed, activity becomes charge-dependent: highly cationic EADs gain broad-spectrum lysis, whereas others lose activity altogether. Unlike Gram-positive bacteria, Gram-negative organisms possess only 1–3 highly conserved PG layers.^64^ This conservation enables both natural and engineered endolysins to function extracellularly with broad host specificity. Their broad activity is linked to their single EAD, which uses strong positive charge and hydrophobic patches to cross the outer membrane without needing to bind a specific bacterial epitope. Examples include LysPA26, which lyses *P. aeruginosa*, *E. coli*, *K. pneumoniae*, and *A. baumannii*, and the endolysins TSPphg and LysAB2, both of which target a broad spectrum of Gram-negative and some Gram-positive bacteria.^65^ Depending on their catalytic specificity, endolysins are classified into muramidases (lysozyme-like enzymes), amidases, endopeptidases, and glucosaminidases, each targeting a unique linkage within the peptidoglycan matrix. Muramidases hydrolyze the β-1,4-glycosidic bond between *N*-acetylmuramic acid (MurNAc) and *N*-acetylglucosamine (GlcNAc), while glucosaminidases cleave the reciprocal GlcNAc–MurNAc linkage. Amidases target the amide bond connecting MurNAc to the l-alanine of the stem peptide, whereas endopeptidases disrupt peptide cross-links that provide mechanical strength to the bacterial cell wall.^66,67^

The catalytic mechanisms of endolysins commonly involve general acid–base catalysis. In muramidase-type endolysins, catalysis proceeds through protonation of the glycosidic oxygen by a conserved glutamate residue, facilitating cleavage of the β-1,4 linkage. A conserved aspartate often acts as a base to activate a water molecule, which performs a nucleophilic attack on the anomeric carbon (C1), leading to bond cleavage *via* an SN1- or SN2-like mechanism depending on enzyme architecture.^68,69^ This strategy closely resembles the classical lysozyme mechanism and underscores the evolutionary conservation of glycoside hydrolase chemistry.

#### 2.1.2 Biological activity, clinical status and comparison between designed and isolated endolysin I from organisms against MDR

The newly characterized endolysin LysSYL exhibited rapid bacteriolytic activity, completely killing planktonic MRSA USA300 within 10 min at 50 µg mL^−1^; at 32 µg mL^−1^ it removed preformed *S. aureus* biofilms (24–72 h). At 500 µg mL^−1^ LysSA52 decreased ∼60% of mixed species *staphylococcal* biofilms; engineered variants like LysKP213 (with a permeabilizer) extended bactericidal activity to MDR Gram-negative pathogens including *Klebsiella*, *Pseudomonas*, and *Acinetobacter*, demonstrating a broadened spectrum.^70^ At the same time, LysAB1245 exhibited dose-dependent killing (>4 log reduction) and biofilm disruption for *P. aeruginosa* and MRSA, while LysPEF1 1/2 reduced MDR *Enterococcus faecalis* biofilms by ∼4.5 log in 2 h. SAL200 reached Phase I human trials in 2017, establishing initial safety and pharmacokinetics.^71^ No subsequent Phase II/III trial results are available, but preclinical evidence supports its bactericidal and anti-biofilm efficacy against MDR *S. aureus*. Preclinical studies have demonstrated that endolysins are promising tools against multidrug-resistant bacteria. LysSYL efficiently disrupts *S. aureus* biofilms, including resistant isolates. Ply113 exhibits strong lytic activity against MRSA and VRE, clearing both mono- and mixed-species biofilms. LysSA52 reduces MRSA biofilm biomass by nearly 60% within 12 hours, and LysPEF1-1/PEF1-2 targets MDR *Enterococcus faecalis*, significantly lowering its biofilm viability.^72^ Additionally, the engineered LysECD7-SMAP demonstrated efficacy and safety in preclinical *in vivo* studies, with clinical-trial protocols under development. The endolysin LysSYL exhibited rapid bacteriolytic activity, completely killing planktonic MRSA USA300 in under 10 min at 50 µg mL^−1^; at 32 µg mL^−1^ it removed preformed *S. aureus* biofilms (24–72 h). At 500 µg mL^−1^, LysSA52 decreased ∼60% of mixed species staphylococcal biofilms; engineered variants like LysKP213 (with permeabilizer) extended bactericidal activity to MDR Gram-negative pathogens including *Klebsiella*, *Pseudomonas*, and *Acinetobacter*, demonstrating broadened spectrum.^70^ At the same time, LysAB1245 exhibited dose-dependent killing (>4 log reduction) and biofilm disruption for *P. aeruginosa* and MRSA, while LysPEF1 1/2 reduced MDR *Enterococcus faecalis* biofilms by ∼4.5 log in 2 h. A study reported that LysKP213, an endolysin from *Klebsiella pneumoniae* phage, retains significant enzymatic activity at extreme temperatures, with 44.4% activity after 20 h at 95 °C and 57.5% after 30 min under autoclave conditions.^73^ Bacteriophage-derived engineered endolysin with a polycationic nonapeptide (PCNP), φSE218_Lys76_L1 PCNP significantly surpasses the native enzyme, eliciting a ∼3 fold reduction in the number of viable Gram-negative cells, whereas the native enzyme exhibited negligible lysis without permeabilizers.^74^ Docking studies further confirm the improvement, showing that the modified endolysin binds peptidoglycan fragments with a higher affinity (−7.19 kcal mol^−1^) than that of the parent enzyme (−6.92 kcal mol^−1^), enabling better outer membrane traversal and enhanced activity on live and heat-inactivated cells. According to a study, the fusion of cecropin A to endolysin (eAbEndolysin) enhanced its antibacterial effect, resulting in a 2–8 fold increase in activity against various MDR *Acinetobacter baumannii* clinical isolates relative to the native enzyme. The study reported that eAbEndolysin had markedly lower MICs (∼15.6–31.2 µg mL^−1^) than those of the parental lysin (∼62.5–125 µg mL^−1^), and corresponding MBC measurements confirmed more efficient bacterial killing.^75^*In vivo* studies showed that eAbEndolysin treatment improved survival in mice during lethal systemic *A. baumannii* infection, with approximately 40% surviving.

### 2.2 DNase I

Deoxyribonuclease I (DNase I, EC 3.1.21.1), isolated from bovine pancreas, is an enzyme used for treating MDR pathogen infections by cleaving the 3′,5′-phosphodiester bond of biofilm-associated extracellular DNA.^76–78^ Biofilms having extracellular DNA (E-DNA) are present in various species such as *Streptococcus mutans*, *Bordetella pertussis*, *Enterococcus faecalis*, *Streptococcus pneumoniae*, *Listeria monocytogenes*, *Campylobacter jejuni* and *Staphylococcus epidermidis*.^79^ E-DNA plays a key role in the attachment and interaction between the biofilm surface and the bacterial membrane. DNase can be paired with any nanoparticle to enhance its effectiveness. For example, it was reported that when carbon monoxide (CO) mesoporous polydopamine nanoparticles (MPDA NPs) were paired with DNase, they proficiently destroyed the biofilms. DNase works against the plasmid DNA; both extracellular DNA and plasmid DNA biofilms resemble each other.

It was reported that DNase I has a molecular weight of 30–40 kDa and has optimum activity at pH 6.5–8, and its activity can be degraded by the addition of EDTA/EGTA in the reaction mixture^80–82^ since it requires calcium and magnesium ions for its activity.^81^ The genome of DNase I with a length of approximately 3.2 kb comprises 9 coding regions and 8 non-coding regions.^83^ The upstream regulatory element (URE) contains a TATA box having “TATAWAW” conserved sequences, and the CAAT box contains “GGCCAATCT” sequences.^84^ DNase I contain 260 amino acids out of which 22 amino acids form a signal peptide.^81^ Many scientists have also found that DNase I plays a role as a digestive enzyme.^85^ The technique used to purify DNase is column chromatography using various types of resin materials like Concanavalin A bound with agarose, DEAE-Sepharose, Sephadex G-75 and Phenyl Sepharose, which is the most efficient resin for DNase purification.^86^ The activity of DNase I was measured by the precipitation and hyperchromicity assay, introduced by the scientist named Kunitz.^87^

A study reported the efficacy of DNase against *Candida albicans* strains (ATCC 96901) by employing the photodynamic therapy (PDT) combination therapy with DNase-I.^88^ PDT involves the destruction of target cells by the formation of reactive oxygen species (ROS) through the excitation of a photosensitizer (PS). When exposed to light, PDT uses PSs to generate ROS, which triggers damage to the cell.^89^ Though PDT works best with the free-floating bacteria rather than the associated biofilm, when biofilms were incubated with DNase and later subjected to PDT, it was found that the strains ATCC 96901, R14, and R70 of fluconazole-resistant *Candida albicans* biofilms showed a tremendous decrease in the E-DNA. Moreover, the viability of cells and the level of water-soluble polysaccharides (WSPs) got reduced, which leads to instability of the ECM.^88^

Another study discussed the effectiveness of DNase against biofilms when combined with a solid lipid nanoparticle encapsulated with anacardic acid and then layered with DNase and chitosan. Chitosan was paired by providing a positive charge to enhance the adhesion of lipid nanoparticles to the negatively charged biofilms.^90^ It was reported that if DNase is used to prevent biofilm formation, a low amount of antibiotic concentration is required. Research stated that biofilm percentage reduction (BPR) of *E. coli* and *S. aureus* strains comes out to be 47–58% when 5 µg mL^−1^ of DNase was pre-treated, and when post-treatment of 10 µg mL^−1^ was given, BPR increases to 73–77%.^91^ In another research, it was found that when high levels of DNase I were taken (1000 µg mL^−1^) with *Helicobacter pylori*, only 50% BPR was achieved. Another report mentioned that for *Listeria monocytogenes* biofilm, the BPR was found to be 50% when 100 µg mL^−1^ of DNase concentration was used.^91^ Later, a study reported the incorporation of Mg^2+^ ions as a cofactor, enhancing the overall efficacy of DNase I by resulting in 75–80% of BPR and a decrease in the concentration of DNase I by 85%. Several studies suggested that when mixed species were used, there was a drastic decline in the BPR as compared to the individual ones. Another study reported a BPR of 36–76% for two *Klebsiella* biofilms. Some other studies reported BPR to be 35% with species *Staphylococcus epidermidis* and *Candida albicans* and 25% with species *Candida albicans* and *Streptococcus gordonii*. It was further reported that DNase isolated from *Vibrio alginolyticus*, which is a marine bacterium, can degrade the *Candida* sp. biofilms.^91^ Nevertheless, there are currently no published studies on the BPR of more than two species.

BPR can be calculated using the following formula:
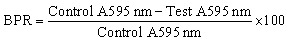


DNase I is used as a biomarker in the field of forensic sciences in criminal and paternity testing.^92^ Moreover, the recombinant form of human DNase I (Pulmozyme) is involved in the treatment of cystic fibrosis patients and asthma patients. It was also reported that pus treated with DNase shows reduced viscosity. Dementia patients are treated with DNase I. DNase is reported to play a role in neutrophil extracellular trap (NET) degradation, a component that traps neutrophils, but sometimes NETs cause damage to the cells by inducing inflammation and exerting some harmful effects that need to be neutralized by DNase I treatment.

#### 2.2.1 Structure and mechanism of action of DNase I

Studies have shown that DNase I (of both bovine and rat origin) is inhibited by *Hypericum* extracts; rutin, in particular, markedly suppresses its enzymatic function, suggesting possible dietary effects and links to male infertility. Catalytically, DNase I displays a striking substrate preference, cleaving dsDNA with an efficiency 100–500-fold higher than that of ssDNA.^93^ Among the known DNA conformations, the B-form represents the most favourable substrate for DNase I, while the Z-form maintains a structural configuration that confers relative resistance to enzymatic hydrolysis.^94^ Human DNase I originates from the DNASE1 gene positioned on chromosome 16p13.3. The gene spans nearly 3.2 kb and comprises 9 exons separated by 8 introns, reflecting a typical multi-exonic structure for secreted enzymes. Single nucleotide polymorphism (6 in number)-based DNASE1 alleles (DNASE11–6) were identified by Yasuda *et al.* Later, 18 missense, 7 nonsense, and 9 indel mutations were reported in the DNASE1 gene, many causing reduced or no enzyme activity.^95^ The catalytic efficiency of DNase I depends on four key residues—Glu78, His134, Asp212, and His252—located within its active site. Several flanking residues—Gln9, Arg41, Tyr76, Arg111, Asn170, Tyr175, and Tyr211—contribute to optimal catalytic geometry and substrate orientation, as evidenced by mutagenesis studies. The enzyme's structural stability is maintained by two conserved cysteines, Cys173 and Cys209, which form a disulfide bond. Interaction with G-actin, which inhibits DNase I, involves four additional residues—Glu13, Tyr65, Val67, and Ala114.^96^ Moreover, two conserved *N*-glycosylation sites at Asn18 and Asn106 are essential for obtaining full enzymatic capability in mammalian systems. Its mechanism is metal-ion dependent, requiring Mg^2+^ or Ca^2+^ to activate a water molecule that serves as the nucleophile. The reaction proceeds through a pentavalent phosphorus transition state, culminating in the cleavage of the P–O bond and the generation of 3′-hydroxyl and 5′-phosphate termini.^97,98^ This metal-assisted nucleophilic substitution is a hallmark of phosphodiesterase activity.

#### 2.2.2 Biological activity, clinical status and comparison between designed and isolated DNase I from organisms

The nuclease DNase I targets extracellular DNA, a scaffold molecule critical for biofilm integrity, thereby weakening the biofilm architecture. *In vitro*, DNase I at 10–50 µg mL^−1^ significantly inhibited biofilm formation and disrupted established biofilms of *P. aeruginosa* and *S. aureus*. When combined with antibiotics (1–32 mg L^−1^ DNase I), the sensitivity of MDR *K. pneumoniae* biofilms to drugs improved markedly, and catheter-associated biofilm infection was largely cleared in an animal model. In a rabbit model of pleural empyema, intrapleural DNase I reduced early biofilm development, lowering bacterial aggregation, matrix formation, and biofilm mass.^99^

Recombinant human DNase I (dornase alfa) remains the clearest clinical success of an enzyme therapy that targets extracellular DNA to disrupt biofilms and mucus: it was approved by the U.S. FDA as Pulmozyme® for cystic fibrosis in 1993 and is established as a nebulized mucolytic agent that improves pulmonary clearance in CF patients.^100,101^ Since approval, investigators have pursued repurposing and new delivery approaches rather than additional broad approvals—small clinical/pilot studies have tested inhaled dornase alfa in acute respiratory syndromes (for example in COVID-19/ARDS) with encouraging safety and signal-finding results but no large definitive indication expansion to date.^102^

Parallel preclinical and translational milestones have shown that DNase I markedly potentiates antibiotics and other antibiofilm enzymes when used as an adjuvant: *in vitro* and animal wound models, DNase I combined with antibiotics or with complementary enzymes (*e.g.*, alginate lyase and proteases) lowers minimal biofilm eradication concentrations and accelerates biofilm clearance and wound healing, and nanoparticle/controlled-release formulations (DNase-loaded SLNs or other carriers) have been reported to improve local efficacy in infected wounds.^103^ Contemporary reviews therefore position DNase I as a validated, regulatory-approved tool (for mucus/DNA clearance in CF) and an active platform for combination therapies against multidrug-resistant biofilm infections—but most applications outside CF remain at the preclinical to early clinical (pilot/repurposing) stage and require larger, indication-specific trials to establish efficacy and safety.^104^ Biofilm architecture is weakened by the nuclease DNase I, which targets extracellular DNA, a scaffold molecule essential to biofilm integrity. *In vitro*, *P. aeruginosa* and *S. aureus* biofilms were disturbed and biofilm development was greatly reduced by DNase I at 10–50 µg mL^−1^. Antibiotics (1–32 mg L^−1^ DNase I) significantly increased the drug sensitivity of MDR *K. pneumoniae* biofilms, and in an animal model, catheter-associated biofilm infection was mostly eliminated.^99^ In a rabbit model of pleural empyema, intrapleural DNase I reduced early biofilm development, lowering bacterial aggregation, matrix formation, and biofilm mass.

### 2.3 Dispersin B

Dispersin B (EC 3.2.1.52), glycoside hydrolase, was characterized for the first time in 2003 from *Aggregatibacter actinomycetemcomitans*, which is a Gram-negative, non-motile periodontopathogenic bacterium.^105,106^ Some scientists have analysed the involvement of genes during biofilm dispersal by creating a transposon library of *Aggregatibacter actinomycetemcomitans* strain (CU1000), and it was found that 5 of the genes were defective during dispersal.^107^ Out of 5 genes, 3 mutant genes were involved in LPS synthesis, another mutant had disruption in the pts1 gene, which regulates sugar transport inside the cell, and the last mutant, JK1023, into which the transposon was inserted, was supposed to code for β-hexosaminidase enzyme.^107^ Later, the gene was named the dsp B gene, which encodes for a 381 amino acid protein.^107^ It is a CAZy-type 20 β-hexosaminidase enzyme that mediates the breakdown of PNAG, *i.e.* poly-β-(1 → 6)-*N*-acetylglucosamine, or PIA, *i.e.* polysaccharide intracellular adhesin.^108^ PNAG is a core component of exopolysaccharide (EPS) of *Staphylococcus epidermidis*, *S. aureus*, *E. coli*, *Klebsiella pneumoniae* and *Acinetobacter baumannii* biofilms and is responsible for the formation of biofilm and microbial virulence as well.^109^ PNAG develops at a very low scale and is closely attached to the cell surface, so it is difficult to extract PNAG. Therefore, for the detection purpose, confocal microscopy is being used with the labelling of F598 human monoclonal antibodies (mAb). A scientist reported that when mAb F598 is labelled with *Yersinia pestis* cells and *Bacillus subtilis*, it leads to a loss in their immunogenicity.^110^ Dispersin B is known to disrupt both biofilms and pellicle formation, due to which the pathogen becomes more vulnerable to antibiotics and immune attack.^111^ Depending upon the substrate properties, dispersin B can perform both exo-glycosidase and endo-glycosidase activity and works efficiently with deacylated PNAG.^107^ A mutant D242 is accountable for dispersin B (DspB_D242N_) endoglycosidase activity that possesses electrostatic interactions.^41^ Along with biofilm disruption, it is also engaged in several other activities like dissociation of free-floating biofilms, removal of established biofilms, weakening of existing biofilms for easier detachment through EDTA, SDS, proteinase K, DNase, and high-pressure water flow, interference during surface adhesion, suppression of hyphal networking, microbial biofilm development, and air–liquid biofilm assembly. Different amino acids like Asp27, Asp183, Glu184, Glu332, Trp216, Trp237, and Trp330 contribute significantly to substrate bond cleavage in the *Acinetobacter baumannii* strain. Literature suggests that oxygen and hydrogen peroxide are responsible for biofilm dispersal.^112^ It was reported that if a mutation was caused in the residue Asp183Asn, it led to 10 000 times reduction in the enzyme activity, while in the case of Glu184Gln, it reduced the activity by 70-fold. Moreover, mutation in Glu332 reduced the activity by 2000 times, and there was 5–2000 fold reduction in enzyme activity in the case of aromatic amino acid residues such as Tyr187, Tyr278, Trp237, and Trp330.^112^

Dispersin B is a single-subunit enzyme having a barrel-like structure with 8 α-helices and 8 parallel β-strands.^107^ The enzyme's active site contains a negative charge due to the presence of acidic molecules. The hexose rings are bound at the 12 Å space created by Trp330 and Trp216 amino acids, while Asp183 and Glu184 remain unaltered across all classes of glycoside hydrolase.^107^ The crystal of dispersin B was formed by the hanging-drop vapour diffusion technique to evaluate the three-dimensional model of the enzyme.^107^ A study reported the biofilm disruption of *Staphylococcus epidermidis* when conjugated with the silver nanoparticle with the N-terminal of dispersin B.^113^ Another study stated that when *Staphylococcus epidermidis* cells were cultured and given dispersin B treatment, it resulted in cell disruption, proving PNAG influence in cell adhesion. One more study in which *E. coli* strains that do not produce PNAG were later treated with PNAG, revealed that upon treatment with dispersin B, the biofilms hydrolyzed.^114^ Another report stated that in *E. coli*, the C-terminus of dispersin B was fused with the MagR protein (iron–sulfur protein present primarily in eukaryotes) to form magnetic nanoparticles. Researchers found that B-MagR, which was loaded onto Fe_3_O_4_/SiO_2,_ was more efficient in eradicating biofilms, rather than the protein alone, because immobilization enhances the stability and can withstand severe temperature conditions.^115^ Dispersin B prevent the biofilm formation of *Staphylococcus epidermis* and *Staphylococcus aureus* when adsorbed onto some polyurethane surface.^116^

Dispersin B is generally layered on some medical equipment to prevent the patients from any kind of wound infection.^103^ In medical device coatings, dispersin B has been incorporated into polymers and hydrogels to create anti-biofilm surfaces.^117^ For example, a study demonstrated that catheters pre-coated with dispersin B substantially reduce bacterial colonization and biofilm development during *in vivo* implantation, thereby reducing the risk of infection and prolonged device functionality.^107^ In wound healing, topical formulations of dispersin B, often delivered *via* hydrogels or nanocarriers, have shown effectiveness in disrupting mature biofilms and accelerating the healing process. This is particularly relevant for diabetic foot ulcers and burns, where biofilm presence severely hampers tissue regeneration.^118^ In oral health, dispersin B has been found in mouth rinses and dental coatings to prevent dental plaque formation, which is essentially a biofilm.^119^ By disrupting bacterial adhesion and biofilm structure, it contributes to better oral hygiene and a reduction in gingivitis and periodontitis.^120^ Dispersin B has been tested in combination therapies with antibiotics such as ciprofloxacin, gentamicin, and rifampicin. These combinations lead to synergistic effects, where the enzymatic removal of biofilm barriers enhances drug diffusion and bacterial susceptibility. This co-administration strategy is considered a promising approach to resensitize methicillin-resistant *Staphylococcus aureus* (MRSA) and biofilm-producing *Escherichia coli* strains.^121^

#### 2.3.1 Structure and mechanism of action of dispersin B

PNAG exists in two primary forms: a positively charged version with ∼15% de-*N*-acetylated GlcNAc, and a zwitterionic subtype distinguished by periodic *O*-succinylation. When PNAG is chemically or enzymatically degraded, or when biosynthetic genes are knocked out, biofilms collapse and virulence diminishes, highlighting PNAG's essential role. According to CAZy, DspB is a member of family 20 glycosyl hydrolases.^108,122^ Structurally, it exhibits a (β/α)8 TIM barrel fold, with a catalytic site predicted to reside within a 13 Å-deep pocket at the barrel's core, enabling effective substrate engagement and catalysis.^123^ In the active site, the GlcNAc residue at the −1 subsite adopts a ^4^E (envelope) conformation, enclosed by a conserved aromatic residue network. This structural arrangement serves to precisely orient the *N*-acetamido oxygen for nucleophilic attack, a key step in glycosidic bond hydrolysis. The catalytic site of DspB features two acidic residues flanking the glycosidic bond:^124^ E184 acts as a general acid to protonate the leaving group oxygen, while the second residue stabilizes the oxazolinium ion intermediate, enabling efficient glycosidic bond hydrolysis. The negatively charged amino acids on the electrostatic surface of DspB form a shallow anionic groove adjacent to the catalytic pocket, which may be important for substrate recognition and orientation, facilitating efficient cleavage of PNAG. Within DspB, residues D147, D245, and E248 are located along the shallow anionic groove and are situated within approximately 15 Å of the catalytic site. This positioning indicates that they may contribute to substrate recognition,^125^ conserved as an acidic residue (Asp or Glu) among GH20 orthologues closely related to DspB. The remaining residues D245 and E248 are situated within a unique α6-helix extension, a feature not observed in any other GH20 enzyme structures. This distinct element may underlie DspB's specialized ability to interact with cationic PNAG analogs.

Its catalytic mechanism involves protonation of the glycosidic oxygen followed by nucleophilic attack by a base-activated water molecule on the anomeric carbon, resulting in glycosidic bond hydrolysis.^126^ Such biofilm-degrading enzymes are increasingly explored as antivirulence agents.

#### 2.3.2 Biological activity, clinical status and comparison between designed and isolated dispersin B from organisms

Evidence indicates that dispersin B is still at a preclinical stage, where it has been shown to increase the susceptibility of *Cutibacterium acnes* biofilms to benzoyl peroxide and tetracycline and to disperse mixed *C. acnes*/*Staphylococcus epidermidis* biofilms. Reports of its ability to boost the effects of various biocides and its PNAG-targeting specificity suggest valuable potential in topical acne management. Despite these trends, its advancement into clinical testing has not yet occurred.^127^

Evidence indicates that dispersin B is still at a preclinical stage, where it has been shown to increase the susceptibility of *C. acnes* biofilms to benzoyl peroxide and tetracycline and to disperse mixed *C. acnes*/*S. epidermidis* biofilms. Reports of its ability to boost the effects of various biocides and its PNAG-targeting specificity suggest valuable potential in topical acne management.^128^ Despite these trends, its advancement into clinical testing has not yet occurred. Experimental data show that treatment with dispersin B in the range of 5–80 µg mL^−1^ significantly reduces biofilm viability, especially when combined with conventional antimicrobials like benzoyl peroxide, even 5 µg mL^−1^ DspB + 0.5% BP led to >6 log reduction in CFU in dual species biofilm of *C. acnes*/*S. epidermidis*.^129^ This suggests that low concentrations (in mg mL^−1^) can significantly disrupt biofilms or sensitize them to antimicrobial agents. Immobilization on Fe_3_O_4_@SiO_2_ nanoparticles allowed DspB MagR to remove over 50% of biofilms, compared to ∼10% by the free enzyme, and raised the optimum temperature from 30 °C to 37 °C, demonstrating superior functional stability and applicability. The fusion of AgBP2 to DspB resulted in ∼2-fold higher catalytic activity, a rise in *S. epidermidis* biofilm clearance from ∼37% to ∼69%, and equivalent biofilm removal at ∼20 fold reduced enzyme concentration.^129,130^

### 2.4 Nanozymes

Nanozymes (EC 1.11.1.7) are nanomaterials which have enzymatic activities, and recently, a therapy based on nanozymes has been used to treat drug-resistant pathogens like Gram-positive *Staphylococcus aureus* and Gram-negative *E. coli*.^131,132^ The therapy utilizes those nanozymes which have characteristics of peroxidase (POD), catalase (CAT) or oxidase (OXD).^133^ POD nanozymes decompose hydrogen peroxide (H_2_O_2_) into reactive oxygen species (ROS), which oxidatively damage proteins, lipids, or nucleic acids and cause irreparable damage.^134^ ROS are very reactive substances that are created from water, H_2_O_2_, and diatomic oxygen (O_2_). These molecules generate oxidative radicals that deteriorate biomolecules and induce apoptosis. The most common PODs used are streptavidin, horseradish peroxidase (HRP) and cytochrome c peroxidase. The Fe_3_O_4_ magnetic nanoparticles resemble the characteristics of HRP that might be utilized in the biosensor preparation.^135^ The oxidase family uses molecular oxygen to oxidize its substrate molecules. Examples include cholesterol oxidase (Cox), alcohol oxidase (AOx), uric acid oxidase (UOx), glucose oxidase (GOx) and lactate oxidase (LOx) that make use of their specific substrates. Nanoparticles like Au, CeO_2_ and MnO_2_ mimic oxidase-like properties. Another class of antioxidant nanozymes includes superoxide dismutase (SOD) and catalase that eliminate ROS since its excessive amount leads to serious side-effects.^136^

Graphdiyne (GYD), molybdenum trioxide (MoO_3_), V_8_C_7_ nanodots (NDs), and ferroptosis (programmed cell death dependent on iron) are examples of nanomaterials that have been used for antibacterial treatment. GYD, which is a carbon allotrope having sp-hybridization, showcases an exceptional structural framework. When it gets doped with boron (B), it becomes B-GYD and mimics the POD activity to produce ROS.^137^ Another nanomaterial is molybdenum trioxide (MoO_3_), which was doped to create a void for the O_2_ molecule, so that the nanomaterial's absorption capacity can be adjusted as per the requirement.^138^ MoO_3_ performs its POD activity by primarily generating O_2_˙, which is very harmful for a bacterial cell as it causes membrane disruption and might cause some lethal mutations, which interfere with different processes and cell death may occur. MoO_3_ can be doped with Au because the Au–MoO_3_ conjugate will produce more free radicals that will help in the eradication of resistant bacteria.^139^ Though the work on Au–MoO_3_ synthesis is not reported as such and remains a significant area for further exploration. There is another V_8_C_7_ nanodot, which is an HRP-like nanozyme that facilitates ROS production by H_2_O_2_ catalysis, and it requires an acidic environment (pH = 5.5) for their functioning. They usually have good absorption efficiency, which photothermally helps in destroying the pathogens.^140^

Another mechanism involved in cell death is ferroptosis, in which cellular death occurs through the lipid peroxidation process, where iron (Fe^2+^) plays an active role in producing ROS, and this whole process is referred to as the Fenton reaction.^141^ A few other methods like photodynamic therapy (PDT), photothermal therapy (PTT) and chemodynamic therapy (CDT) have been explored to treat drug-resistant pathogens, but nanozyme-based therapy is excellent because it provides better stability, is cost-effective and is very easy to process.^142^ Moreover, temperature significantly influences the activity of nanozymes by producing ROS in abundance. Nanozymes are extensively used in serological testing and biosensing devices.^143^

#### 2.4.1 Structure and mechanism of action of nanozymes

Their catalytic behaviour is largely governed by redox-active metal centers exposed on the nanoparticle surface such as Fe^2+^/Fe^3+^ or Ce^3+^/Ce^4+^, which undergo reversible oxidation–reduction cycling during catalysis. As a result, nanozymes commonly mediate redox reactions and radical-based pathways, including the generation of reactive oxygen species such as hydroxyl radicals (˙OH) and superoxide anions (O_2_˙^−^). Many nanozymes also display peroxidase-like activity, where hydrogen peroxide is reduced *via* surface-facilitated electron transfer mechanisms. The defining chemical hallmark of nanozyme catalysis is therefore surface-mediated single-electron transfer (SET), rather than the classical acid–base or nucleophilic mechanisms characteristic of natural enzymes.^144,145^

#### 2.4.2 Biological activity, clinical status and comparison between designed and isolated nanozymes from organisms

Nanozymes are nanomaterials that exhibit intrinsic enzyme-like catalytic activities, such as peroxidase, oxidase, catalase, superoxide dismutase (SOD), hydrolase, and lactonase-like functions. Their biological activity arises from surface redox centers, unsaturated metal coordination sites, oxygen vacancies, and quantum size effects, which collectively enable electron transfer, substrate adsorption, and bond cleavage reactions.^146^ In biological systems, nanozymes have demonstrated potent antimicrobial activity, reactive oxygen species (ROS) regulation, anti-biofilm effects, anti-inflammatory responses, and anticancer properties. For example, Fe_3_O_4_, CeO_2_, MnO_2_, and Pt-based nanozymes modulate ROS homeostasis by mimicking peroxidase/catalase/SOD activities, thereby influencing oxidative stress pathways relevant to infection, neurodegeneration, and cancer. Compared with natural enzymes, nanozymes show higher operational stability under extreme pH, temperature, and ionic conditions, making them particularly attractive for *in vivo* and translational applications.^147^

Although nanozymes are not yet approved as standalone enzyme replacement therapies, several have progressed into preclinical and early clinical evaluation, primarily as antimicrobial agents, antioxidants, biosensing components, and cancer therapeutics. Iron oxide nanozymes are the most clinically advanced, benefiting from prior regulatory approval of iron oxide nanoparticles as MRI contrast agents, which significantly lowers translational barriers. Cerium oxide nanozymes have entered clinical trials for oxidative stress-related disorders including retinal degeneration and inflammatory diseases due to their regenerative redox cycling (Ce^3+^/Ce^4+^). In antimicrobial therapy, nanozymes are being evaluated as alternatives or adjuvants to antibiotics to combat multidrug resistance (MDR) by disrupting biofilms and inducing localized ROS-mediated bacterial killing. Overall, nanozymes are predominantly used in preclinical to Phase I stages, with ongoing optimization focused on biosafety, biodegradability, pharmacokinetics, and target specificity.^148,149^

Designed (synthetic) nanozymes and enzymes isolated from organisms differ fundamentally in their origin, catalytic behavior, stability, and translational potential. Designed nanozymes are engineered nanomaterials including metal-, metal oxide-, carbon-, and metal–organic framework (MOF)-based systems, whose enzyme-like activity arises primarily from surface-driven redox reactions,^150^ Lewis acid–base interactions, and electron transfer mediated by exposed metal centers. In contrast, natural enzymes are derived from biological systems such as plants, microbes, and animals, and possess highly evolved three-dimensional active sites composed of specific amino acid residues and cofactors that govern catalysis. As a result, nanozymes typically exhibit broader and tunable substrate specificity, whereas natural enzymes display remarkable substrate selectivity and catalytic precision. From a stability perspective, nanozymes are highly robust, maintaining activity across wide ranges of pH, temperature, and storage conditions, while isolated enzymes are often sensitive to environmental fluctuations and susceptible to denaturation or proteolytic degradation. Production and scalability also favor nanozymes, as they can be synthesized reproducibly at low costs with minimal batch-to-batch variation, whereas enzymatic preparations usually require complex purification procedures and may suffer from variability.^151^ In terms of biocompatibility, nanozymes generally exhibit low immunogenicity, whereas natural enzymes can elicit immune responses in clinical settings. Clinically, most nanozymes remain at preclinical or early clinical stages, while many natural enzymes, such as l-asparaginase and DNase I, are already approved and widely used in therapy. Importantly, the design flexibility of nanozymes—allowing precise control over size, shape, composition, and surface functionalization—far exceeds that of biological enzymes, which are constrained by their native structures. Overall, while designed nanozymes offer robustness, scalability, and multifunctionality, enzymes isolated from organisms excel in catalytic efficiency and molecular specificity. Consequently, contemporary research increasingly emphasizes hybrid and bio-inspired strategies, including enzyme–nanozyme composites, to integrate the precision of natural enzymes with the durability and adaptability of nanomaterials.^150,152^

### 2.5 Lysostaphin

**Fig. 3:**
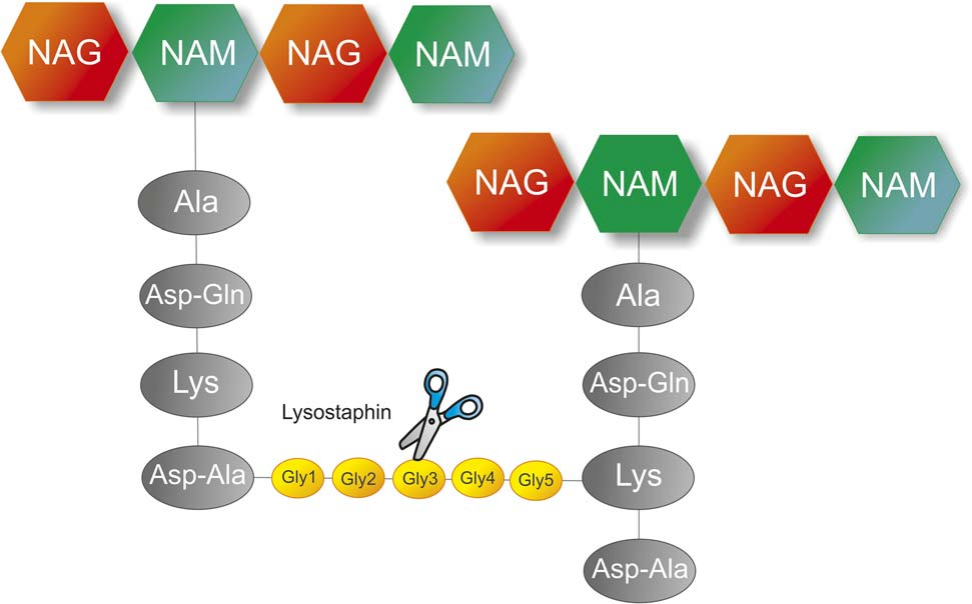
Diagram of the lysostaphin bacteriolytic activity against *S. aureus*. The enzyme specifically cleaves between the pentaglycine peptides present between two NAM molecules.

Lysostaphin (EC 3.4.24.75), a class IIIa bacteriocin, which is a metalloenzyme requiring zinc for its functioning, has a molecular mass of 27 000 Da and contains 246 amino acids.^153–155^ Lysostaphin was discovered in 1964 from *Staphylococcus simulans biovar staphylolyticus* that cleaves the 2nd and 3rd or 3rd and 4th glycine molecules in a penta-glycine-peptide present between NAM molecules of the peptidoglycan layer (Fig. 3).^156,157^ Lysostaphin acts specifically in the treatment of drug-resistant pathogen *Staphylococcus aureus.*^43^ The genome of *S. aureus* contains a few genes, such as FmhB, which code for monoglycine, FemA, which codes for triglycine, and FemB, which codes for pentaglycine. If any of these genes were mutated, then it might cause a loss in the integrity of the peptidoglycan layer. Lysostaphin unveils a greater potency than that of antibiotics like vancomycin and penicillin. Lysostaphin disintegrates the cells in both the stationary phase and the growth phase.^43^ The N-terminal domain (residues 1–137) of lysostaphin, which belongs to the M23 peptidase family and contains residues H32, D36, and H115, performs catalytic function, *i.e.*, hydrolyzes the glycine pentapeptide present between the NAM molecule of the peptidoglycan layer. The C-terminal domain (residues 154–246) of the SH3b family, which is in charge of the enzyme's adheres to the bacterial cell and contains a linker molecule (residues 138–153).^158^ More research is needed on the engineering of lysostaphin and its formulation to promote the efficacy, stability, safety, and delivery efficiency.

The bacteriolytic activity of lysostaphin can be determined using a turbidity assay. A bacterial suspension culture is prepared in a buffer, followed by the addition of lysostaphin enzyme to break the bacterial cells, and then the OD at 550 nm is measured at different time frames. Once the cells get lysed, they become unable to scatter the light, which ultimately leads to a decrease in the OD of the suspension culture. This defines the bacteriolytic activity, *i.e.*, the amount of enzyme which is required to reduce the OD min^−1^.^159^ When a high concentration of enzyme is used, the graph is sigmoidal in shape and at a low concentration, a straight-line graph is obtained. For each and every reaction, there should be three different concentrations of enzyme that have to be used to determine the rate of turbidity reduction.

The catalytic assay of lysostaphin can be performed by pentaglycine peptide, based on the hydrolysis of pentaglycine into diglycine and triglycine chains that give free amino groups, which, when reacted with ninhydrin, give a colour change that can be measured spectrophotometrically. First, pentaglycine is dissolved in boiling water at a temperature above 95 °C and then the buffer and NaCl solution are added, followed by the addition of lysostaphin, and then divided into different aliquots to incubate them at 37 °C. All the aliquots are taken out at different time intervals, such as 0, 8, 24, 32, and 48 hours, and later kept at −70 °C. Now, to find out how much of the pentaglycine is hydrolysed, aliquots are kept at a temperature above 95 °C for 10 min with the addition of ninhydrin solution and then the OD is measured in a 96-well plate at 595 nm. The reported optimum pH for catalytic activity is 8.5.^159^

Lysostaphin is not thermally stable at temperatures above 50 °C, so it might cause some inconvenience during long-term storage of lysostaphin and also in the course of preserving food items containing lysostaphin.^158^ For that, scientists have conducted various studies by making some mutations to render it thermostable. One of the studies is the incorporation of disulfide bridges in the zinc docking site, which can enhance the lysostaphin stability against high temperatures.^160^ Later, it was found that the stability of lysostaphin was increased by approximately 2.5-fold when treated for 30 min at a temperature above 60 °C, after the gene alteration of glycine polymer at the C-terminal. They replaced the S–S bond with the existing metal ion present in the molecule.^161^ The milk exposed to the *Staphylococcus aureus* can be treated by the addition of lysostaphin, which decreases the toxicity of the pathogen.^43^

In the clinical case of a patient suffering from MRSA-pneumonia, when 500 mg of lysostaphin was injected, the symptoms of the disease, including low blood pressure, erythema, and increased heart rate, were controlled.^43^ A report stated that BMP-2 (Bone Morphogenetic Protein-2)-loaded lysostaphin hydrogels, which were synthetically produced, helped in the eradication of *Staphylococcus aureus*.^162^ In another study, lysostaphin was encapsulated within a nanoparticle like polylactic-*co*-glycolic acid (PLGA) to overcome loss in its catalytic activity, maintaining its stability and preventing it from developing any immunogenic response that might cause inflammation or any negative effects.^163^ Moreover, it was reported that they have modified lysostaphin to form its derivative F12, which became immunotolerant by substituting 14 amino acids. F12 enters the human body without provoking any immune response and can be used several times to treat MRSA infections. Upon performing the MIC assay, it was found that F12 was 20-fold more effective than lysostaphin.^164^ Another study suggested that to enhance the antibacterial activity, the lysostaphin catalytic domain was integrated with the bacteriocin E50-52, which was produced from the bacteria *Enterococcus faecium* and has ∼40 amino acids with the ability to withstand extreme thermal and pH conditions. It was proven that E50-52 downregulates the toxicity of MRSA infections, nosocomial infections, *E. coli*, *Salmonella* spp., *Staphylococcus* spp., *Campylobacter jejuni*, and *Shigella dysenteriae*.^165^ In extremely high or low affinity of the enzyme, the observed activity was very low, but in moderate affinity, the activity of lysostaphin increased when the catalytic binding domain was analyzed through X-ray crystallography and nuclear magnetic resonance. Moreover, some studies suggested that enzyme's lytic activity can be enhanced by increasing the positive charge and the concentration of NaCl.^159^

Advanced methods facilitate convenient purification and low-cost production of recombinant lysostaphin (rLysostaphin). The very first rLysostaphin was obtained in 1987 from *E. coli*.^166^ For the purification purpose, it was typically labelled with a His-tag. Lysostaphin was expressed in *Bacillus subtilis* with a yield of 91 mg L^−1^ and in *Lactococcus lactis* producing 300 mg L^−1^. In addition to this, lysostaphin stops the growth of staphylococcal microbes in food to prevent the food from poisoning when exposed to *Staphylococcus aureus*, *Staphylococcus carnosus*, and other species of staphylococci. Lysostaphin has been formulated many times to obtain different products for treating infections. In the past few years, it was formulated as a cream/gel-based product, a chitosan oil-in-water cream, a gel with the formulations of hydroxypropyl methylcellulose, and a petroleum-based cream, which was reported to reduce the MRSA infection in rats. Lysostaphin can be entrapped into a hydrogel, or encapsulated into chitosan bone cement, or coated on titanium discs, all of which can treat bone and joint deformities.^167^ In the case of skin and wound infections, lysostaphin immobilization can be done covalently onto cellulose fibres or cross-linked into the matrix of a mixture of compounds (collagen, chitosan, and gelatin). In recent years, the encapsulation of lysostaphin and vancomycin into exosomes attached with the mannose residue was successfully done, which eradicates the MRSA infectious cells.^168^ Moreover, lysostaphin is coated onto the surface areas frequently utilized in public spaces to prevent the spread of *Staphylococcus aureus*.^166^ Lysostaphin is combined with an antibiotic molecule to ensure that *Staphylococcus aureus* is targeted at different regions and does not gain resistance easily.

#### 2.5.1 Structure and mechanism of action of lysostaphin

The class III bacteriocin lysostaphin is produced as a 493-residue pre-proenzyme comprising: (i) a 23-residue signal peptide, (ii) a 224-residue tandem repeat region, (iii) a Zn^2+^-bearing catalytic domain, (iv) a 16-residue linker, and (v) a 92-residue CWT domain.^169^ The removal of the N-terminal signal sequence and tandem repeats during proteolytic processing results in the mature ∼28 kDa enzyme, designated as Lss. Its CAT domain carries the conserved HXXXD and HXH motifs characteristic of M23 metallopeptidases.^170^ Several M23 Zn^2+^-dependent endopeptidase structures are now available, among which are Lss, zoocin A, LytM, and LytU from *S. aureus*. The Lss structure indicates that the Zn^2+^-coordinated active site sits within a substrate-binding groove, with His279, Asp283, and His362 acting as the coordinating ligands.^171^ Four uncharacterized loops (1–4) within the Lss-CAT domain form the architecture of the substrate-binding groove, suggesting functional roles in substrate recognition. Two loop residues—Tyr270 (loop 1) and Asn372 (loop 4)—were identified as highly conserved and capable of interacting with the pentaglycine substrate. Docking analysis showed that Tyr270 and Asn372 engage the carbonyl oxygen atoms of pentaglycine through their side-chain functional groups—Tyr270 *via* its hydroxyl (2.5 Å) and Asn372 *via* its amide NH_2_ (2.8 Å)—supporting their proposed roles in substrate interaction.^172^

Lysostaphin is a Zn^2+^-dependent metalloprotease that specifically cleaves Gly–Gly peptide bonds within the pentaglycine cross-bridges of *Staphylococcus aureus* peptidoglycan. The catalytic zinc ion polarizes the peptide carbonyl, while an activated water molecule attacks the amide bond to form a tetrahedral intermediate, which collapses to release cleaved peptide products.^171,173^ This metal-assisted hydrolysis exemplifies targeted antibacterial specificity.

#### 2.5.2 Biological activity, clinical status and comparison between designed and isolated lysostaphin from organisms

For biofilm eradication assays (24 h preformed biofilms), treatment with diopside lysostaphin containing ∼0.52 µg mL^−1^ of adsorbed lysostaphin resulted in “almost complete” biofilm elimination (by crystal violet staining). In planktonic + biofilm viability (CFU reduction) assays: minimal bactericidal concentration (MBC-P for planktonic cells) from the diopside lysostaphin composite was 0.20 mg mL^−1^ diopside—∼1.04 µg mL^−1^ lysostaphin; MBC for biofilm-embedded cells (MBC-B) was 0.10 mg mL^−1^ diopside—∼0.52 µg mL^−1^ lysostaphin.^174^

Recent preclinical studies indicate that lysostaphin could become a viable therapeutic agent against MDR staphylococcal infections. Researchers have encapsulated lysostaphin in biodegradable polylactic-*co*-glycolic acid nanoparticles, which maintain its activity and enable effective eradication of biofilms and intracellular *S. aureus in vitro*.^163,175^ An engineered, modular version of lysostaphin shows enhanced bioavailability and a significantly prolonged half-life, resulting in much stronger intracellular antimicrobial activity. *In vivo* studies further support its potential: lysostaphin-functionalized titanium implants markedly reduced MRSA-associated osteitis in minipigs, demonstrating effectiveness against implant-associated infections. A study by Zhao *et al.*^176^ directly compared wild-type and electrostatically engineered lysostaphin. The engineered form showed markedly faster MRSA lysis (shorter time-to-50% OD drop, higher lysis rates), altered cell-wall binding, and enhanced *in vivo* therapeutic performance, despite similar MIC values. The engineered variant significantly increased survival in mice with bacteraemia (*P* = 0.029), indicating improved enzymatic processivity and interaction with its target over the native lysostaphin. A study has recently engineered a nano-assembled lysostaphin by immobilizing it onto lysine-rich polypeptide nanoparticles, which achieved about 12-fold greater intracellular activity against MRSA than the native enzyme.^177^ Furthermore, the engineered enzyme possessed a prolonged circulation half-life and greatly enhanced shelf-stability, mitigating major limitations of the native enzyme for therapy.

### 2.6 Alginate lyase

**Fig. 4:**
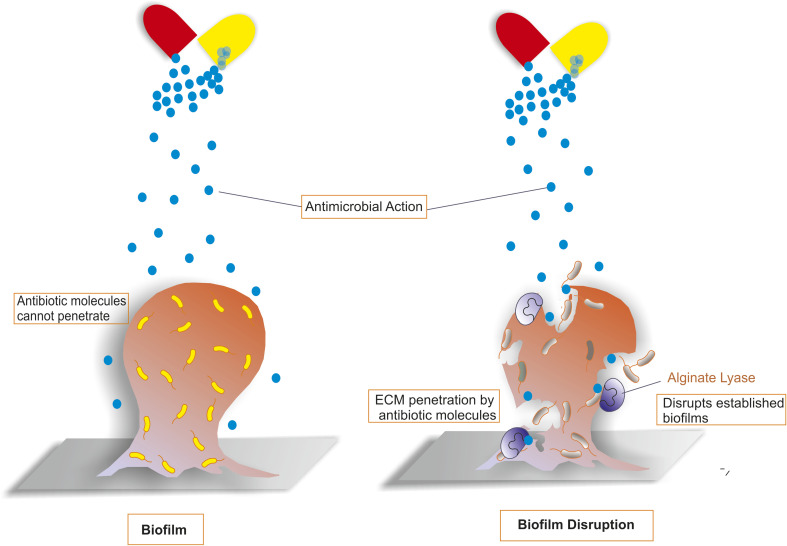
An illustration of how alginate lyase works. Antibiotics cannot pass through the membrane when they come into contact with biofilms that are encased in alginate extracellular matrix (ECM). However, when alginate lyase acts on the biofilm, it degrades the alginate ECM, which makes it easier for antibiotics to pass through the membrane and cause biofilm degradation.

Alginate lyase (EC 4.2.2.3) degrades the biofilms by cleaving the 1–4 glycosidic bond through a beta-elimination reaction.^178,179^ As shown in Fig. 4, antibiotics are unable to enter the extracellular matrix of biofilm in the absence of alginate lyase, but in the presence of alginate lyase, the biofilm gets disrupted, and antibiotic molecules can invade and destroy bacteria. Biofilms are protective coverings around *Pseudomonas aeruginosa* in defence against any antibiotic or drug molecule. Biofilms are made up of alginate, which is a linear polysaccharide that comprises the units of β-d-mannuronate (M) and α-l-guluronate (G) and are linked in 3 different ways: (polyM), (polyG), and (polyM/G/G).^180^ In β-D-M, at position C-2 or C-3, alginate is esterified with an acetyl group, and the degree of acetylation may vary depending on the source of carbon and the specific strain.^181^ Alginate can increase the infection by adhering to the host cell membrane. The different sources reported for alginate lyases are marine bacteria *Flammeovirga* sp. (NJ-04), *Serratia marcescens* (NJ-07), and *Saccharina japonica*.^182^

The degradation of alginate occurs in various steps. First, the carboxylic acid containing a negative charge is removed (RCOO^−^) to lower the p*K*_a_ value of H-5. Later, a proton from C-5 is removed by a base molecule that gives a negative charge on C-5, which is then localized onto a carbonyl oxygen group (C=O), leading to the formation of an enolate ion (CO^−^). In the last step, double bond formation occurs between carbon positions 4 and 5 by the transfer of an electron and further leads to hydrolysis of the glycosidic bond. The two mechanisms by which alginate lyase catalyses the β elimination reaction are dependent on Ca^2+^ and based on histidine or tyrosine residues.^183^

Twelve polysaccharide lyase (PL) families (PL5, 6, 7, 14, 15, 17, 18, 31, 32, 34, 36, and 39) were identified from the database of carbohydrate-active enzymes (CAZy).^184^ Some lyases cleave extrinsically to produce alginate monosaccharides and disaccharides, while some have intrinsic activity to produce oligosaccharides. There are various diseases associated with biofilms, such as ventilator-associated pneumonia (VAP), cystic fibrosis (CF), and chronic obstructive pulmonary disease (COPD). It was reported that the alginate lyase QY101 of the bacteria *Vibrio* failed to suppress the biofilm activity of a strain FRD1 of *Pseudomonas aeruginosa* obtained from the sample of a CF patient.^185^ Scientists have found that the alginate lyase (AlyP1400) isolated from *Pseudoalteromonas*, a marine bacterium, disrupts the alginate purified from mucoid *Pseudomonas aeruginosa* strain CF27. Extreme temperatures were found to cause the enzyme to lose its catalytic activity when detected utilizing immunofluorescence staining with the alginate-targeted antibody.^186^ It was also reported that alginate lyase Smlt1473 from *Stenotrophomonas maltophilia* K279a strain can hydrolyse poly-ManA (d-mannuronic acid), which is a main component of alginate present in *Pseudomonas aeruginosa*.^187^

Another study confirms that if the MIC value comes out to be greater than the concentration of the antibiotic, then there is overproduction of alginate by *Pseudomonas*. As reported by two scientists, Hatch and Schiller, the ability of positively charged antibiotics such as aminoglycosides and polymyxin B to kill the pathogen can be obstructed by a negatively charged entity, alginate.^188^ The operon machinery contains 12 different types of genes (algA, algB, algC, algD, and so on) which are involved in the alginate synthesis. The enzyme alginate lysate is transcribed through the algL gene, which is present between algG and algF.^189^

The enzyme alginate lyase serves multiple applications across various fields, such as in the development of alginate oligosaccharide (AOS).^190^ Second, it is widely used in ethanol production. Moreover, alginate plays a crucial role in the food industry, solidifies food items by the chelation of calcium ions present in them, and slows down the absorptivity of different nutrients by forming a gel-like structure, and similarly, it holds the glucose moiety and regulates the blood glucose level in the body. Alginate regulates the ABA production in strawberries, assisting in increasing their shelf life.^191^ Moreover, alginate is widely used in the treatment of water pollution. Alginate has wide-ranging applications in carbon recycling by converting large chunks of matter into smaller particles.^192^ Both alginate lyases and lactonases remain at the preclinical stage, as no human clinical trials have been reported to date. Alginate lyases have shown promising antibiofilm activity against *Pseudomonas aeruginosa*, improving antibiotic susceptibility and disrupting biofilms *in vitro*, as well as in wound-dressing and cystic fibrosis infection models. Lactonases, which disrupt bacterial quorum-sensing signals, have demonstrated reduced biofilm formation, lowered virulence factor production, and enhanced antibiotic efficacy *in vitro* and in animal or *ex vivo* models.

#### 2.6.1 Structure and mechanism of action of alginate lyase

Alginate lyases act *via* β-elimination to cleave the 4-*O*-glycosidic linkages, producing oligosaccharides that bear a 4-deoxy-L-*erythro*-hex-4-enopyranosyluronic acid at the non-reducing end. Alginate lyases catalyze the β-elimination of 4-*O*-glycosidic bonds *via* a multi-step mechanism. The reaction begins with the neutralization of the substrate's carboxyl group through a salt bridge.^193^ Next, a Brønsted base abstracts the proton at C5, while a Brønsted acid donates a proton to stabilize the transition state. At last, electrons from the carboxyl group are transferred to cleave the 4-*O*-glycosidic bond, forming a C4=C5 double bond.^194^ The resulting products are unsaturated alginate oligosaccharides bearing 4-deoxy-L-*erythro*-hex-4-enopyranosyluronic acid at the non-reducing terminus. The stereochemistry of the products depends on the residue type: syn-configuration occurs in M residues and anti-configuration in G residues, influencing biofilm polysaccharide structure and degradability. The catalytic mechanism of alginate lyases can be generally categorized into two types—His (or Tyr)/Tyr elimination and metal ion-assisted elimination—based on C5 carboxyl neutralization and the Brønsted acid–base chemistry involved. Only a subset of PL6 alginate lyases adopt the metal-ion-assisted mechanism, as shown in AlyGC, where a Ca^2+^ ion within the active site neutralizes the negative charge of the substrate's C5 carboxyl group. In contrast, alginate lyases from PL5, PL7, PL14, PL15, PL17, and PL18 families operate through a His (or Tyr)/Tyr-mediated β-elimination mechanism. PL5, PL14, and PL18 alginate lyases utilize Tyr/Tyr elimination, where Tyr acts as both the proton acceptor and donor.^195^ PL7 and PL15 enzymes use His/Tyr elimination, with His as the acceptor and Tyr as the donor. Most PL17 alginate lyases utilize the His/Tyr elimination mechanism, similar to PL7 and PL15. Alg17c, however, represents a notable exception, employing Tyr450 as the catalytic base and Tyr258 as the catalytic acid. The functional distinction of two Tyr residues differentiates the catalytic mechanism of some alginate lyases from the Tyr/Tyr mechanism observed in PL5, PL14, and PL18. Beyond Tyr and His, other residues can modulate the activity. In Aly36B from PL36, a Lys residue replaces Tyr, demonstrating how alternative amino acids can sustain catalytic efficiency.^196^ Interestingly, AlyF from the PL6 family utilizes an H_2_O-assisted mechanism. The water molecule stabilizes and positions the C5 carboxyl group, with Arg293 acting as the catalytic base and Lys272 as the general acid, working together to complete the enzymatic reaction. The structural characterization of alginate lyases has revealed four main folds. The PL5 (α/α)*n* toroid fold comprises antiparallel α-helices, forming a tunnel-like active site, as in A1-III. PL6 and PL31 β-helix enzymes, such as BcAlyPL6, contain N-terminal domains for substrate binding and C-terminal domains forming the catalytic cleft. The β-jelly roll fold, adopted by PL7, 14, 18, and 36, forms a globular shape with a catalytic pocket in the inner concave sheet. PL15, 17, and 39 multidomain enzymes fuse toroid and β-sheet domains, forming unique pockets crucial for exolytic catalysis, exemplified by AlyA3 and Atu3025. Within the PL7 family, three conserved regions—SA3 (RXEXR), SA4 (YXKAGXYXQ), and SA5 (QXH)—have been proposed as determinants of substrate preference. Typically, the presence of QVH correlates with polyM specificity, and QIH with polyG or polyMG activity.^197^

Alginate lyases differ mechanistically from hydrolases by catalyzing β-elimination rather than hydrolysis. These enzymes cleave β-1,4 linkages in alginate by abstracting a proton from C5, leading to the formation of an unsaturated Δ4,5-uronic acid product. This non-hydrolytic elimination mechanism is critical for biofilm dispersal in *Pseudomonas* species.^198^

Finally, quorum-quenching lactonases hydrolyze the ester bond of acyl-homoserine lactone signalling molecules, thereby disrupting bacterial communication. These enzymes often employ a metal-dependent nucleophilic acyl substitution mechanism involving water activation, tetrahedral intermediate formation, and ring opening of the γ-lactone.^199^ Such enzymes hold promise as anti-virulence therapeutics.

#### 2.6.2 Biological activity, clinical status and comparison between designed and isolated alginate lyases from organisms

Alginate lyases applied at 6–25 µg mL^−1^ significantly inhibited *P. aeruginosa* biofilm formation and disrupted established biofilms (up to ∼60% biomass reduction), when immobilized on a bacterial cellulose wound dressing. Certain PL7 family alginate lyases maintained their activity even in the presence of metal ions (Fe^2+^, Zn^2+^, Mn^2+^, *etc.*) in settings that mimicked the lung environment of cystic fibrosis, such as high metal ion concentrations.^200^

Since there have been no documented human clinical studies, both lactonases and alginate lyases are still in the preclinical stage. Alginate lyases have shown promising antibiofilm activity against *Pseudomonas aeruginosa*, improving antibiotic susceptibility and disrupting biofilms *in vitro*, as well as in wound-dressing and cystic fibrosis infection models. Lactonases, which disrupt bacterial quorum-sensing signals, have demonstrated reduced biofilm formation, lowered virulence factor production, and enhanced antibiotic efficacy *in vitro* and in animal or *ex vivo* models.^201^

The immobilization of Aly08 on low molecular-weight chitosan nanoparticles significantly enhanced its thermal stability, retaining ∼76.8% activity after 1 h at 37 °C, whereas the free enzyme's activity dropped by ∼60% in just 40 min. Upon incubation at 45 °C, immobilization conferred superior thermal stability to the enzyme, resulting in significantly higher residual activity relative to the free enzyme. The immobilized form also retained over 60% activity after six reuse cycles, whereas the free enzyme was unusable after a single cycle.^202^

### 2.7 Lactonase

Lactonases are widely present in bacteria, fungi and plants and are characterized based on their substrate specificity; those having broad specificity are metallo-β-lactamases (MBL) lactonases, for instance, GcL (*Geobacillus caldoxylosilyticus*).^45^ The other one, which has long acyl chains, is phosphotriesterase-like lactonases (PLL) such as SsoPox, W263I, and paraoxonases (PONs). One of the MBL lactonases is YtnP-lactonase (Lac), which was isolated from *Stenotrophomonas maltophilia*, a Gram-negative bacterium found mainly in soil, water, and the human body.^45^ YtnP-Lac aids in the treatment of *Pseudomonas aeruginosa* MMA83 strain. YtnP-Lac is also a quorum quenching (QQ) enzyme, which deals with the MDR pathogens by inhibiting the quorum sensing (QS) molecules.^203^ QS is the process that occurs in bacterial species. Whenever there is a modulation in the bacterial cell population, they interact with each other and cause some changes in their gene expression by recruiting some signalling molecules, namely autoinducers such as *N*-acyl-homoserine lactones (AHLs), and gain resistance against drug molecules.^204^ To break this communication, they develop some means, like the use of QS inhibitors or QQ enzymes. There are three varied groups in which QQ enzymes are classified: oxidoreductases, lactonases and acylases.^205^ Among all these, lactonase is the one that facilitates overcoming MDR bacteria by cleaving the ester bond of the AHL molecules, which consequently disturbs the mechanism of working of QS by opening their lactone ring. The examples of AHLs are LrsL (Labrenzia, red sea bacterium lactonase), AidC from *Bosea* sp., and Parathion Protein Hydrolase (PPH) lactonase from *Mycobacterium tuberculosis*.^45^

Four pathways of *Pseudomonas aeruginosa*'s QS system have been identified: rhamnolipid-based (RHL), LasI-based (las), integrating (IQS), and Pseudomonas Quinolone Signal (PQS). The PQS and IQS systems have not yet been thoroughly investigated.^206^ The rhl system contains an RhlR transcriptional regulator, while the las system contains a LasR transcriptional regulator. Some AHLs have been reported to remarkably inhibit the activity of 3-oxo-C12-HSL against the LasR system of *Pseudomonas aeruginosa*, C9 (nonanoyl)-CPA acts upon *Serratia marcescens*, Zingerone on *Pseudomonas aeruginosa*, and *N*-(3-oxododecanoyl)- or *N*-(3-oxotetradecanoyl)-L-HSL on *Aeromonas hydrophila* by creating a communication gap between QS receptor molecules and the AHLs.^207^ Several studies reported that there are some bacterial lactonases, including AiiA (isolated from *Bacillus thuringiensis*), AiiB (from *Agrobacterium tumefaciens*), AidC, and AaL (from *Alicyclobacter acidoterrestris*).^208^ It has also been reported that when QQ enzyme works in combination with any antibiotics like ciprofloxacin, it leads to the elimination of the pathogen.^209^

There are some artificially made analogues termed furanones that play a role in QS inhibition.^210^ The most prominent source of furanone is the red algae *Delisea pulchra*, which interferes with the motility of *Serratia liquefaciens* and also suppresses the expression of *E. coli* during flagella synthesis.^211^ A scientist named Lonn-Stensrud stated that the furanones having bromophenol as a functional group can prevent the biofilm formation by *Staphylococcus epidermidis*.^212^ Some other products that are considered QS inhibitors are patulin, sulfur compounds, penicillanic acid and halogenated furanones.^213^ To treat cystic fibrosis patients, the macrolide antibiotics could be a useful option, and the compound 4-nitropyridine-*N*-oxide which is present in garlic, inhibits the QS virulent genes, while the catechin obtained from *Combretum albiflorum* suppresses the virulence gene production.^214^ To the best of our knowledge, there is very limited research done on the mechanism of regulatory molecules to inhibit QS to counteract the *P. aeruginosa* pathogen.

#### 2.7.1 Structure and mechanism of action of lactonase

Lactonases constitute the majority of AHL-degrading enzymes, and they are broadly classified into four families: metallo-β-lactamases, paraoxonases, α/β-hydrolase lactonases, and phosphotriesterase-like lactonases. The metallo-β-lactamase class of AHL lactonases, which are extensively studied, possess a Zn^2+^-binding HXHXDH motif essential for catalyzing the hydrolysis of acyl-homoserine lactones including AiiA from *Bacillus* sp. 240B1. In vertebrates, especially mammals, paraoxonases (PON1, PON2, and PON3) are highly conserved. PON2 displays robust AHL inactivation and arylesterase activity, while PON1 and PON3, although less effective at AHL degradation, metabolize various substrates such as organophosphates, arylesters, and γ- and δ-lactones. Transgenic expression of human PON1 in *Drosophila* attenuated OC12-HSL-mediated virulence of *P. aeruginosa*, decreased oxidative stress markers, and altered the gut microbial population. The α/β-hydrolase family of lactonases are distinguished by a conserved nucleophile–histidine–acid catalytic triad, where the acidic component is either Asp or Glu. This category includes enzymes such as AidH isolated from *Ochrobactrum* sp. T63, AiiM from *Microbacterium testaceum* StLB037, and JydB from *Rhodococcus* sp. BH4. As members of the amidohydrolase superfamily, PLLs possess wide substrate specificity. Certain PLL enzymes, commonly called paraoxonases, efficiently hydrolyze organophosphate substrates like paraoxon, whereas others demonstrate a weaker activity toward phosphotriesters but robustly degrade various lactones. Well-studied examples of PLLs comprise QsdA from *Rhodococcus erythropolis*, GKL sourced from *Geobacillus kaustophilus*, and the GsP enzyme derived from *G. stearothermophilus*.

From a chemical standpoint, lactonase catalysis is most often mediated by a metalloenzyme mechanism, frequently involving a binuclear metal center such as Zn^2+^/Zn^2+^, Zn^2+^/Fe^2+^, or Co^2+^, characteristic of the metallo-β-lactamase superfamily. In this mechanism, the coordinated metal ions polarize the carbonyl group of the ester (lactone) bond and activate a bridging hydroxide or water molecule, increasing its nucleophilicity. The activated hydroxide performs a nucleophilic attack on the electrophilic carbonyl carbon of the lactone ring, leading to the formation of a tetrahedral intermediate, which subsequently collapses to generate the corresponding ring-opened hydroxyacid. This catalytic process follows a nucleophilic acyl substitution pathway and does not involve redox cycling or radical intermediates; the electron flow is restricted to bond rearrangements during ester hydrolysis. Chemically, lactonases are therefore defined by metal-assisted nucleophilic hydrolysis, resulting in irreversible inactivation of quorum-sensing signals and effective quorum-quenching activity.^215^

#### 2.7.2 Biological activity, clinical status and comparison between designed and isolated lactonases from organisms

Lactonases are quorum-quenching enzymes that catalyze the hydrolytic cleavage of the lactone ring in *N*-acyl homoserine lactones (AHLs), which are key quorum-sensing (QS) signaling molecules in many Gram-negative bacteria. From a biochemical perspective, lactonase activity typically involves a nucleophilic attack on the ester bond of the homoserine lactone ring, leading to ring opening and irreversible inactivation of the signal molecule. Most characterized lactonases belong to the metallo-β-lactamase (MBL) superfamily or the paraoxonase (PON) family and require divalent metal ions such as Zn^2+^, Co^2+^, or Mn^2+^ for catalysis. Biologically, lactonases attenuate bacterial virulence without directly killing bacteria by disrupting QS-regulated processes, including biofilm formation, toxin secretion, motility, and antibiotic tolerance. This anti-virulence mechanism reduces selective pressure for resistance development and has shown efficacy against clinically relevant pathogens such as *Pseudomonas aeruginosa*, *Acinetobacter baumannii*, and *Burkholderia* spp., highlighting lactonases as promising tools against multidrug-resistant (MDR) infections.^199,216,217^

In a study by Muras *et al.*, purified Aii20J was applied at 20 µg mL^−1^ to *in vitro* periodontal biofilm cultures, which corresponded to ∼10×, the minimal concentration required for AHL degradation in 24 h.

Despite strong preclinical evidence supporting their anti-virulence and anti-biofilm potential, lactonases have not yet reached late-stage clinical trials as standalone therapeutics. Most lactonase-based interventions remain in the preclinical phase, evaluated *in vitro* and in animal infection models. Challenges limiting clinical translation include enzyme instability in physiological environments, short systemic half-life, potential immunogenicity, and difficulties in targeted delivery. To overcome these barriers, recent strategies involve protein engineering, immobilization on nanoparticles, encapsulation in hydrogels, and fusion with delivery carriers, which significantly enhance stability, bioavailability, and *in vivo* efficacy. Especially, lactonases are also being explored as medical device coatings, wound dressings, and adjunct therapies combined with antibiotics, where regulatory pathways may be less stringent than systemic drug approval. Overall, while no lactonase has yet received regulatory approval for human use, their favorable safety profile and resistance-suppressing mechanism continue to drive translational research.^218,219^

Lactonases isolated from organisms, including bacteria, archaea, and mammals, are products of natural evolution and possess highly specific three-dimensional active sites optimized for AHL recognition and hydrolysis. These enzymes exhibit high catalytic efficiency and substrate selectivity but often suffer from limited operational stability, sensitivity to pH and temperature variations, and susceptibility to proteolytic degradation, which restricts their therapeutic applicability. In contrast, designed or engineered lactonases, developed through rational design, directed evolution, or nanozyme-inspired approaches, offer enhanced robustness, broader substrate tolerance, and improved activity under physiological and pathological conditions. Engineered lactonases can be tailored by modifying metal-binding residues, active-site geometry, or surface charge to expand AHL specificity and increase resistance to denaturation. Furthermore, designed systems—such as lactonase-mimicking nanozymes or enzyme-nanomaterial hybrids—enable scalable production, reduced batch variability, and integration with multifunctional platforms for targeted delivery and imaging. While natural lactonases currently dominate fundamental QS research, engineered and bio-inspired lactonases are emerging as more practical candidates for clinical translation, combining the molecular precision of biological enzymes with the durability and design flexibility of synthetic systems.^220,221^

## 3 Current advances in enzyme therapy for MDR pathogens

The global rise in MDR pathogens exerts a pressing challenge to modern medicine. Conventional antibiotics, once considered the cornerstones of infectious disease management, are losing their effectiveness due to widespread resistance mechanisms, such as efflux pumps, enzymatic degradation, and target site modifications. In this context, enzyme therapy has emerged as a promising and innovative strategy to combat MDR pathogens. Unlike small-molecule drugs, enzymes act as biological catalysts that can selectively degrade essential bacterial structures, neutralize virulence factors, or inactivate resistance-conferring biomolecules. Their high specificity, biodegradability, and ability to function in synergy with conventional antimicrobials make them attractive alternatives or adjuncts to traditional treatments. Enzymes are built from the ground up instead of being altered from naturally occurring proteins in *denovo* enzyme design, which is a paradigm change. This approach relies on a deep understanding of protein folding, active-site architecture, transition-state stabilization, and catalytic residue positioning.^222^ Early successes in *de novo* enzyme design demonstrated that artificial enzymes could catalyze reactions such as Kemp elimination and retro-aldol reactions, which are rare or inefficient in nature.^223,224^ These studies established fundamental design principles, including precise geometric placement of catalytic residues and optimization of hydrogen-bond networks to stabilize reaction intermediates.

Rational enzyme design complements *de novo* strategies by leveraging structural, mechanistic, and evolutionary knowledge of existing enzymes to guide targeted mutations.^225^ Structure-based mutagenesis has been widely used to improve substrate specificity, catalytic efficiency, and thermal stability, particularly in industrial and pharmaceutical enzymes.^226^ Semi-rational approaches, which combine rational design with limited directed evolution, have proven especially effective in overcoming the limitations of purely predictive methods while minimizing experimental screening efforts.^227^

Computational methods form the backbone of contemporary enzyme design and deserve further elaboration. Tools such as molecular dynamics simulations, quantum mechanics/molecular mechanics (QM/MM) modeling, and energy minimization algorithms enable accurate prediction of enzyme-substrate interactions and reaction energetics.^228^ Software platforms like Rosetta have played a pivotal role in both *de novo* enzyme creation and redesign of existing scaffolds.^229^ More recently, machine learning and artificial intelligence-based approaches have emerged as powerful tools for predicting protein structure and function, significantly accelerating the enzyme design pipeline.^230^

Beyond methodology, the expanding applications of designed enzymes highlight the importance of this field. Engineered enzymes are increasingly used in synthetic biology for pathway optimization, in medicine for enzyme replacement therapies and prodrug activation, and in green chemistry to replace environmentally harmful chemical catalysts.^231^ Designed enzymes also hold promise in precision medicine, where tailored catalytic functions may be developed to address disease-specific metabolic imbalances.

In conclusion, enzyme design—particularly *de novo* design—represents a transformative area of biotechnology that warrants a more comprehensive discussion. Expanding this section to include foundational principles, computational advances, and real-world applications would greatly enrich the manuscript and provide readers with a clearer understanding of the current state and future potential of enzyme engineering.

### 3.1 Development of phage-derived lysins

Bacteriophage-derived endolysins have gained significant attention for their ability to hydrolyze bacterial cell wall peptidoglycan, leading to rapid cell lysis. These enzymes are particularly effective against Gram-positive pathogens such as *Streptococcus pneumoniae*, *Staphylococcus aureus*, and *Enterococcus faecalis*.^232,233^ Recent advances in protein engineering have expanded their activity spectrum to Gram-negative bacteria by adding outer membrane-permeabilizing domains. Recombinant lysins such as CF-301 (Exebacase) are already undergoing clinical trials, marking a significant milestone in translational enzyme therapeutics.^234,235^ Recently, a study has identified two endolysins, ElyA1 and ElyA2 (members of the GH108-PG3 family), encoded within the genomes of bacteriophages Ab1051Φ and Ab1052Φ, respectively.^236^ Their muralytic activity against multidrug-resistant (MDR) clinical isolates—*Acinetobacter baumannii*, *Pseudomonas aeruginosa*, and *Klebsiella pneumoniae*—was evaluated using a turbidity reduction assay. The minimal inhibitory concentrations (MICs) for endolysin, colistin, and their combination were determined, and the antimicrobial efficacy was validated through time–kill curve analysis.^236^

ElyA1 exhibited broad activity, effectively targeting all 25 tested strains of *A. baumannii* and *P. aeruginosa*, as well as 13 of 17 *K. pneumoniae* strains. In contrast, ElyA2 showed no detectable activity against these isolates. Specifically, combining ElyA1 with colistin resulted in a significant reduction in the colistin MIC for all strains except *K. pneumoniae*. These synergistic effects were confirmed *in vivo* using *Galleria mellonella* survival assays and murine skin and lung infection models. The combination of colistin at one-quarter MIC with ElyA1 (350 µg) enhanced bactericidal activity in both *in vitro* and *in vivo* experiments.^236^ This strategy has the potential to lower the required therapeutic dose of colistin in clinical practice, thereby reducing associated toxicity while maintaining efficacy against MDR pathogens.

### 3.2 Biofilm-degrading enzymes

Biofilms confer high tolerance to antibiotics and are a major cause of chronic infections. Glycoside hydrolases are a diverse group of enzymes that target specific polysaccharides within microbial biofilms, thereby disrupting their structural integrity and enhancing the effectiveness of antimicrobial therapies. One of the most well-studied enzymes is dispersin B (DspB), a member of glycoside hydrolase family 20 (GH20), which hydrolyzes poly-β-1,6-*N*-acetyl-d-glucosamine (dPNAG) *via* a substrate-assisted mechanism and possesses both endo- and exo-glycosidase activities.^237^ DspB is highly effective in dispersing mature biofilms of both Gram-positive and Gram-negative bacteria, and an engineered variant, DspBE248Q, exhibits greater activity than the wild type. It has been commercialized as a wound gel (dispersin B®) by Kane Biotech Inc. Another enzyme, PgaB, containing a CAZy GH153 glycoside hydrolase domain, cleaves dPNAG and disrupts mature biofilms of *Bordetella pertussis*, *Staphylococcus epidermidis*, and *Escherichia coli*. Similarly, alginate lyases, such as those from *Flavobacterium multivorum* and marine sources like AlyP1400, degrade alginate in *Pseudomonas aeruginosa* biofilms, enhance antibiotic penetration, and downregulate efflux pump genes, thereby potentiating tobramycin activity.^238^ Other glycoside hydrolases include PslGh (GH39), which inhibits *P. aeruginosa* biofilm initiation and disrupts young biofilms, and PelAh, derived from the Pel operon, which targets Pel polysaccharides and enhances both antibiotic and immune-mediated killing.^239^ Additional enzymes, such as amylases (GH13), cellulases, levanase SacC, and inulinases, degrade amylose, cellulose, levan, and inulin, respectively, with demonstrated antibiofilm activities.^240^ Fungal biofilms are also targeted by (1,3)-β-glucanases, Sph3h (GH135), and Ega3 (GH114), which effectively disrupt galactosaminogalactan (GAG)- and β-glucan-dependent *Aspergillus fumigatus* and *Candida albicans* biofilms. Collectively, glycoside hydrolases represent promising enzymatic strategies to disperse biofilms, restore antimicrobial susceptibility, and support host immune clearance.^241^

Proteases are important biofilm-disrupting enzymes that target extracellular proteins and adhesins within the biofilm matrix, thereby impairing biofilm stability and promoting bacterial clearance. Proteinase K, a broad-spectrum serine protease with excellent thermostability and wide pH tolerance, is effective in both preventing new biofilm formation and dispersing mature biofilms across a variety of bacterial strains. Similarly, trypsin, a pancreatic serine protease that cleaves peptide bonds on the carboxyl side of lysine and arginine, has been shown to degrade biofilms formed by diverse Gram-positive and Gram-negative species.^242^ Pepsin, an aspartate endopeptidase with specificity for hydrophobic residues such as phenylalanine and leucine, reduces the biomass of preformed *Pseudomonas aeruginosa* and *Enterococcus faecalis* biofilms.^243^ Several proteases are secreted by *Staphylococcus aureus* and act as critical modulators of biofilm physiology. Aureolysin, a secreted metalloprotease, suppresses biofilm initiation and disperses established *S. aureus* biofilms, while V8 serine protease (SspA) degrades the biofilm-associated protein (Bap) to inhibit *Staphylococcus epidermidis* biofilms. Staphopain A (ScpA) and Staphopain B (SspB), both cysteine proteases, regulate biofilm stability, with ScpA directly dispersing *S. aureus* biofilms, whereas SspB modulates biofilm formation through cleavage of the antimicrobial peptide LL-37, reducing host-mediated inhibition.^244^ In addition, Spl proteases (SplABCDEF) contribute to virulence by degrading surface-associated proteins, with their deletion enhancing biofilm development. Other bacterial species also produce biofilm-disrupting proteases. SPRE from *Streptococcus mutans* disperses preformed biofilms by cleaving adhesin P1, while SpeB from *Streptococcus pyogenes* disrupts biofilms at multiple stages, facilitating bacterial dissemination. Finally, peptidase M16, a metalloprotease from *Microbacterium* sp., efficiently disperses *S. aureus* biofilms at low concentrations and synergizes with kanamycin, underscoring its therapeutic potential.^245^

Deoxyribonucleases (DNases) are enzymes that degrade DNA and play crucial roles in the regulation of biofilm formation and dispersal, as well as in therapeutic applications. DNase I is a well-known pancreatic endonuclease that digests both single-stranded and double-stranded DNA.^81^ It is particularly effective at preventing biofilm initiation and disrupting newly formed biofilms, though it is less efficient against mature ones. Recombinant DNase I (rhDNase) has been clinically applied in cystic fibrosis patients to reduce mucus viscosity and limit bacterial infection. In *Vibrio cholerae*, two secreted nucleases, Xds and Dns (VcEndA), contribute significantly to biofilm regulation. Xds, a Mg^2+^-dependent nuclease from the PF03372 protein family, and Dns, a member of the endonuclease I superfamily, both degrade circular and linearized DNA within biofilms.^246^ The deletion of either nuclease gene results in enhanced biofilm formation, highlighting their regulatory functions. Another clinically relevant enzyme is streptodornase (varidase), a mixture of four DNases produced by *Pseudomonas aeruginosa*, which effectively disrupts preformed *P. aeruginosa* biofilms and has been used to treat focal infections. Additionally, NucB, a ββα metal-dependent nuclease from marine *Bacillus licheniformis*, disperses mature biofilms by degrading extracellular DNA and has shown efficacy against bacterial strains isolated from chronic rhinosinusitis. Collectively, these DNases demonstrate both ecological significance in microbial communities and potential therapeutic value.^180^

### 3.3 Enzyme-antibiotic synergy

Combining enzymes with existing antibiotics offers a dual-action approach—enzymes degrade structural or protective components of pathogens, while antibiotics target essential metabolic pathways.^247^ Lysins paired with beta-lactams have demonstrated improved bactericidal activity even against methicillin-resistant *S. aureus* (MRSA).^248^ Such synergy can reduce required antibiotic doses, lowering the risk of toxicity and further resistance development.^249^ The growing threat of multidrug-resistant tuberculosis (MDR-TB) demands innovative therapeutic approaches that can overcome the limitations of conventional antibiotics.^250^ In this context, a novel nano-enzybiotic strategy was developed by immobilizing the mycobacteriophage-derived Lysin B (LysB) enzyme onto a rifampicin-loaded UiO-66 metal–organic framework (Rif@UiO-66) nanocomposite for enhanced inhaled anti-TB therapy.^251^ UiO-66 nanoparticles, synthesized under mild conditions, served as a safe and stable drug delivery vehicle, allowing efficient encapsulation of rifampicin and surface immobilization of LysB. The resulting LysB/Rif@UiO-66 nanocomposite demonstrated remarkable antibacterial potency against *Mycobacterium smegmatis*, a nonpathogenic tuberculosis model. Minimum inhibitory concentration (MIC) assays revealed that LysB/Rif@UiO-66 achieved a 16-fold reduction in MIC compared to free rifampicin, confirming a strong synergistic effect. Computational docking further validated the cooperative interaction between rifampicin, LysB, and UiO-66, highlighting the mechanistic basis of synergy. Importantly, biodistribution studies showed that the nanocomposite provided a 5.31-fold higher drug concentration in the lungs than that of free rifampicin, while *in vitro* and *in vivo* toxicity assessments confirmed its excellent biocompatibility and reduced hepatotoxicity. By enabling targeted pulmonary delivery, LysB/Rif@UiO-66 enhances drug bioavailability, minimizes systemic side effects, and offers a means to bypass resistance mechanisms associated with single-drug therapy.^251^ This nano-enzybiotic platform demonstrates the therapeutic potential of combining phage-derived enzymes with nanotechnology-based drug carriers to strengthen conventional antibiotics. Overall, the LysB/Rif@UiO-66 nanocomposite represents a promising step toward next-generation anti-TB treatments, streamlining rifampicin dosing, improving efficacy, and addressing the urgent need for safe and effective therapies against MDR-TB.

### 3.4 Targeted delivery systems

Recent advances in nanotechnology have improved enzyme delivery to infection sites. Nanocarriers such as liposomes, polymeric nanoparticles, and dendrimers protect enzymes from degradation, prolong circulation time, and allow controlled release.^252^ Targeted systems using antibodies or peptides as ligands ensure enzyme accumulation at the infection site, thereby enhancing the therapeutic efficiency while minimizing systemic exposure. The most significant obstacle in effective cancer therapy is the emergence of MDR during chemotherapy. Cancer cells can adapt and survive repeated cycles of conventional chemotherapeutic agents, eventually developing resistance that limits drug efficacy, and along with that, many hampering pathways like Warburg effect, pyruvate metabolism, alanine, aspartate and glutamate metabolism, histidine metabolism and more pathways play important roles.^253^ This resistance not only reduces treatment success but also leads to cancer recurrence and metastasis, leading to poor prognosis and high mortality.^254^ Combination drug therapy has been widely explored to overcome MDR, yet synthetic chemotherapeutics are often associated with severe side effects and systemic toxicity. As a result, increasing attention has been paid to natural compounds derived from traditional medicines, particularly traditional Indian medicine (TIM), for their low toxicity and ability to sensitize cancer cells for treatment. Mechanisms underlying MDR include drug efflux mediated by ATP-binding cassette (ABC) transporters, inhibition of programmed cell death, and the pro-survival role of autophagy, all of which reduce drug accumulation and cytotoxic effects in cancer cells.^255^ TIM provides bioactive compounds that can modulate these pathways and help reverse resistance. However, poor solubility, limited bioavailability, and low targeting efficiency restrict the clinical utility of many natural products. Here, nanotechnology offers a transformative solution. Novel nano-drug delivery systems can precisely control drug release, enhance solubility, and selectively target cancer cells, thereby improving the pharmacokinetics and therapeutic outcomes of TIM-based combination therapy.^256^ Passive targeting strategies exploit the enhanced permeability and retention (EPR) effect, while advanced delivery vehicles such as liposomes, polymeric nanoparticles, mesoporous silica nanoparticles, and microemulsions improve drug stability and accumulation at tumor sites. Active targeting approaches further engineer nanocarriers with transferrin, folic acid, or low-density lipoprotein (LDL) to enhance selective uptake by cancer cells. The integration of TIM with allopathic therapies *via* nanotechnology offers synergistic advantages. Nano-TIM formulations not only improve drug solubility and penetration but also enhance the pharmacokinetic profile of conventional drugs, reduce adverse effects, and increase therapeutic index.^257^ Importantly, these systems hold the potential to control the expansion of drug resistance by delivering TIM bioactives alongside chemotherapeutics in a single nanoplatform. In conclusion, MDR remains a central challenge in cancer management, but nano-enabled TIM-combination therapies provide a promising strategy to overcome drug resistance, minimize toxicity, and improve clinical outcomes. By bridging traditional medicine and nanotechnology, it is possible to design innovative therapeutic systems that suppress resistance mechanisms, enhance efficacy, and ultimately transform cancer care.

### 3.5 Enzyme engineering and directed evolution

Enzyme engineering has rapidly evolved as a transformative field, driven by site-directed mutagenesis and directed evolution, which have significantly enhanced enzyme stability, substrate specificity, and catalytic efficiency. Additional modifications such as PEGylation further improve pharmacokinetics, prolong circulation time, and reduce immunogenicity, paving the way for “next-generation” enzymes with optimized therapeutic profiles.^258^ A particularly intriguing concept in this context is catalytic promiscuity, the ability of a single enzyme to catalyze multiple mechanistically distinct reactions. This property, central to evolutionary adaptation, is being deliberately harnessed by researchers to design novel enzymes capable of performing new-to-nature transformations. Strategies often involve stabilizing or repurposing reaction intermediates in active sites, followed by directed evolution to fine-tune reactivity, efficiency, and selectivity. Directed evolution—targeted artificial evolution of DNA sequences—has become a cornerstone not only in biocatalysis but also in tackling urgent clinical problems such as antimicrobial resistance. While *in vitro* mutagenesis has been the dominant approach for generating mutant libraries, it is labor-intensive and biased toward certain mutations. *In vivo* mutagenesis addresses many of these limitations but remains underexplored experimentally. Excitingly, recent phage-based continuous evolution systems have eliminated intermediary screening steps, dramatically increasing throughput.^259^ Importantly, such methods can be applied to re-sensitize resistant pathogens, including fungi, thereby extending the therapeutic lifespan of existing drugs. Beyond therapeutic use, biocatalyst engineering is reshaping industrial chemistry by enabling sustainable, enantioselective, and cost-effective synthesis of fine chemicals and pharmaceuticals. Random, semi-random, and computational redesign approaches have already produced highly efficient enzymes under industrial conditions. A notable example is the optimization of the old yellow enzyme family, demonstrating the synergy of enzyme design, evolution, and process optimization. Collectively, these advancements highlight how enzyme engineering—integrating catalytic promiscuity, directed evolution, and process optimization—is shaping the future of medicine and industrial biotechnology.^260^

## 4 Key challenges in enzyme therapy for MDR pathogens

The emergence of MDR pathogens has necessitated novel therapeutic approaches beyond traditional antibiotics. Enzyme therapy—using bacteriophage-derived lysins, depolymerases, or engineered hydrolases—offers a promising strategy due to its ability to directly degrade bacterial cell walls or biofilm components. However, despite promising preclinical results, translation into clinical settings faces several challenges in delivery, immunogenicity, manufacturing, and regulatory approval.

### 4.1 Stability and shelf-life

Enzymes lose activity due to heat, pH shifts, moisture, and proteolysis, and hence, recent work focuses on making them storage- and clinic-ready. Advanced lyophilization with optimized lyoprotectants (*e.g.*, sugars that increase 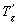) traps proteins in a rigid glass, cutting aggregation and preserving activity during long-term storage and transport.^261^ Dry-powder inhalation formats are gaining traction for respiratory infections: spray-dried protein/nanoformulations show improved stability and room-temperature handling when process stresses are controlled.^262^ Encapsulation/immobilization in metal–organic frameworks (MOFs) such as ZIF-8/UiO-66 protects enzymes from denaturation and proteolysis, boosting thermal and pH resilience and enabling co-delivery with antibiotics.^263,264^ On the protein side, engineered thermostable endolysins and chimeras retain potency after stress exposure, expanding shelf-life without cold chain. Together, formulation science (lyophilization and spray drying), smart carriers (MOFs), and protein engineering (thermostable variants) are converging to deliver enzyme therapeutics that are stable on the shelf, robust in transit, and active *in vivo*—a prerequisite for scalable anti-MDR deployment.^265^

### 4.2 Delivery mechanisms

Efficient delivery of therapeutic enzymes to infection sites remains one of the most formidable challenges, especially in systemic infections and under biofilm-associated conditions. Enzymes often face degradation or clearance before reaching their targets, while Gram-negative bacteria present an additional barrier due to their impermeable outer membranes. Recent studies have explored nanoparticle-based delivery systems such as liposomes and polymeric nanoparticles, which can encapsulate enzymes and protect them from host defenses.^266^ For example, a study demonstrated that endolysins conjugated to cationic polymers achieved enhanced penetration into *Pseudomonas aeruginosa* biofilms, reducing bacterial burden in murine lung infection models.^267^ Another promising approach is modular enzyme engineering, where catalytic domains are fused with targeting peptides or cell-penetrating motifs. For instance, engineered lysins carrying outer membrane-permeabilizing peptides have shown significant bactericidal activity against *Acinetobacter baumannii*, a notorious MDR pathogen.^268^ These strategies highlight the growing potential of precision delivery platforms for overcoming structural barriers to enzyme activity.

### 4.3 Immunogenicity and safety

Foreign proteins, particularly those of bacteriophage or microbial origin, are prone to immune recognition, which can neutralize their effects or cause inflammatory responses. This remains a major limitation for chronic or repeated treatments. Research has turned towards strategies such as PEGylation, which not only prolongs circulation half-life but also reduces immunogenic epitopes. For example, a report showed that PEGylated endolysins retained enzymatic activity while eliciting markedly lower antibody titers in preclinical models.^269^ Other approaches include epitope masking and protein humanization, in which immunodominant sequences are computationally redesigned to resemble human proteins. Synthetic biology platforms now allow “stealth lysins” with minimized immune recognition, maintaining antimicrobial activity without triggering strong adaptive responses.^270^ Importantly, long-term safety studies are still limited, and further evaluation in primate models or early-phase human trials will be essential.

### 4.4 Large-scale production and cost

Manufacturing therapeutic enzymes at pharmaceutical grade remains technically demanding. Production requires high-yield expression systems, stringent purification and stability testing, all of which drive up costs. Current research focuses on improving expression platforms such as engineered *E. coli* strains, *Pichia pastoris* (yeast), and plant-based expression systems.^271^ A study in *Metab. Eng.* demonstrated that glycoengineered *P. pastoris* strains could produce endolysins at 10-fold higher yields while maintaining stability, providing a more scalable and cost-effective approach.^272^ Additionally, cell-free protein synthesis (CFPS) has emerged as a novel strategy for rapid, on-demand production of therapeutic enzymes. CFPS platforms eliminate the need for living host systems, reducing variability and accelerating scale-up.^273^ Although still costly, ongoing innovations in energy-efficient cell-free systems may bridge the gap between laboratory success and industrial-scale feasibility.

### 4.5 Regulatory and clinical validation

Unlike antibiotics, enzyme therapeutics do not have well-established regulatory frameworks, making clinical translation more complex. Regulatory agencies such as the FDA and European Medicines Agency (EMA) require long-term safety data, standardized dosing protocols, and reproducible efficacy metrics.^274^ Clinical trial design remains particularly challenging, as traditional endpoints for antibiotics may not fully capture the unique pharmacodynamics of enzyme-based therapies. Recent progress is encouraging: the first clinical trials of phage lysins (*e.g.*, Exebacase against *Staphylococcus aureus*) have demonstrated safety and efficacy in early-phase studies, paving the way for broader acceptance.^275^ Still, systematic evaluation of combination therapies, resistance development, and pharmacokinetics will be critical for regulatory approval. Furthermore, international harmonization of guidelines for biologics will accelerate clinical adoption.

## 5 Future directions in enzyme therapy research

Enzyme therapy for MDR pathogen infections has moved from theoretical promise to experimental validation, with several lysins, depolymerases, and engineered catalytic proteins entering preclinical and clinical pipelines. Despite encouraging results, challenges remain in delivery, immunogenicity, scalability, and regulatory approval. Addressing these issues requires innovative approaches that combine advanced biotechnology, computational design, and global collaboration. Recent research highlights several promising directions for future enzyme therapy development.

### 5.1 Advanced protein engineering

The next generation of therapeutic enzymes will probably be highly customized to specific pathogens and resistance mechanisms. Advances in protein engineering, fuelled by artificial intelligence (AI) and machine learning (ML), now enable the prediction of beneficial mutations and rational design of catalytic sites. Tools such as AlphaFold2 and Rosetta have accelerated structural modelling, making it possible to fine-tune enzyme-substrate interactions with unprecedented precision. For instance, a study engineered *Streptococcus*-targeting lysins using deep mutational scanning, identifying mutations that enhanced catalytic efficiency while improving thermostability. Similarly, computational redesign has been applied to create variants of Exebacase (CF-301), the first phage lysin tested in human clinical trials, to broaden its activity spectrum and increase serum stability. Another emerging direction is the design of synthetic enzyme scaffolds, where catalytic residues are embedded into modular backbones optimized for stability and solubility. By integrating AI-driven predictions with directed evolution, researchers can rapidly iterate enzyme variants, reducing development timelines and tailoring therapies to specific infection niches, such as biofilm-associated infections or intracellular pathogens.

### 5.2 Multifunctional enzymes

Pathogens often rely on multiple structural defenses including peptidoglycan layers, capsules, and biofilms. Engineering multifunctional enzymes with dual or multiple catalytic domains represents a powerful approach to overcome these barriers. Recent studies have demonstrated the potential of chimeric lysins that combine peptidoglycan hydrolase domains with biofilm-degrading enzymes such as DNases or polysaccharide depolymerases. A report described a fusion enzyme targeting *Pseudomonas aeruginosa* biofilms, where the DNase domain facilitated matrix degradation, enabling the lysin to penetrate and lyse bacterial cells effectively. Multifunctional enzymes may also reduce the likelihood of resistance, as pathogens would need to simultaneously mutate multiple structural components to evade killing. Additionally, the fusion of cell wall-degrading domains with antimicrobial peptides (AMPs) has shown synergistic effects, enhancing bactericidal potency. Going forward, modular enzyme design platforms will allow the assembly of tailored multifunctional constructs, expanding the range of treatable MDR pathogens.

### 5.3 Smart delivery platforms

One of the most pressing challenges in enzyme therapy is efficient delivery to infection sites, especially in systemic or biofilm-associated infections. Recent advances in nanotechnology pave the way for smart delivery systems that release enzymes in response to infection-specific cues such as pH, redox potential, or bacterial enzymes. For example, researchers have developed pH-responsive liposomes encapsulating lysins, which remain stable in circulation but release their payload in the acidic environment of infected tissues. Similarly, enzyme-loaded polymeric nanoparticles have been engineered to degrade selectively in the presence of bacterial proteases, ensuring targeted release. A study reported the use of magnetically guided nanocarriers for lysin delivery to infected bone tissue, significantly improving therapeutic outcomes in a murine osteomyelitis model. Hydrogel-based systems are another promising platform, particularly for wound infections. Injectable hydrogels embedded with lysins and biofilm-degrading enzymes have demonstrated sustained, localized release, effectively clearing biofilms of *Staphylococcus aureus* in animal models. These smart platforms minimize systemic exposure, reduce immunogenicity, and enhance precision targeting.

### 5.4 Combination with host-directed therapies

Enzyme therapy does not need to function in isolation. Combining it with host-directed therapies (HDTs) could yield synergistic benefits by both enhancing immune defenses and directly eliminating pathogens. For instance, pairing lysins with cytokine therapies such as interferon-gamma (IFN-γ) has been shown to enhance macrophage-mediated clearance of intracellular bacteria. Similarly, immunomodulatory peptides that modulate inflammatory pathways may help mitigate tissue damage during infection while enzymes target the pathogens directly. Recent research highlights the potential of checkpoint inhibitors and Toll-like receptor agonists to boost innate immune responses, which could complement enzyme-mediated bacterial killing. Importantly, such combinations may reduce the likelihood of relapse and help control chronic infections where immune evasion is common, such as in tuberculosis or fungal biofilm infections. By aligning enzyme activity with host immunity, researchers envision a dual-pronged strategy that improves therapeutic outcomes and reduces reliance on traditional antibiotics.

### 5.5 Enzyme libraries and personalized medicine

The rise of personalized medicine in oncology and infectious disease management is now extending to enzyme therapy. Building large libraries of engineered enzymes targeting diverse bacterial cell wall structures, resistance enzymes, and virulence factors could enable rapid, tailored treatment strategies. Recent advances in high-throughput screening and microfluidic platforms allow thousands of enzyme variants to be evaluated against clinical isolates in parallel. When coupled with rapid diagnostics such as CRISPR-based pathogen detection, clinicians could quickly identify the causative pathogen and select the most effective enzyme or enzyme combination. For example, a study demonstrated the feasibility of combining whole-genome sequencing of resistant *Klebsiella pneumoniae* with a library of tailored depolymerases to guide precision therapy. Such approaches not only improve efficacy but also minimize off-target effects and preserve commensal microbiota. The vision is a personalized enzyme pharmacy, where bespoke or pre-optimized enzymes can be matched to the patient's infection profile within hours, dramatically changing the landscape of infectious disease management.

### 5.6 Regulatory harmonization and global access

Despite scientific progress, regulatory and accessibility issues remain major bottlenecks. Current regulatory frameworks are designed for small-molecule antibiotics, and there is limited precedent for biologics like enzymes. To accelerate translation, global regulatory harmonization will be essential. Encouragingly, FDA and EMA have initiated discussions on streamlined approval pathways for non-traditional antimicrobials including lysins and phage-derived enzymes. Recent clinical trials of Exebacase have provided valuable insights into trial design, endpoints, and safety assessments. However, consistent guidelines and international collaboration will be critical to avoid duplication and delays. Equally important is ensuring affordable global access. Low- and middle-income countries (LMICs) bear a disproportionate burden of multidrug-resistant (MDR) infections, yet enzyme treatments are currently costly to produce and scale, restricting accessibility and widening health inequities. Advances in recombinant expression systems—such as yeast, plant, and cell-free platforms—may reduce costs, but investment in technology transfer and manufacturing infrastructure is needed. Initiatives similar to the COVID-19 vaccine global access model could provide a template for equitable enzyme therapy distribution.

## 6 Conclusion

A new, cutting-edge method for treating infections brought on by various drug-resistant organisms is enzyme therapy. Although there are some enzymes such as urokinase, lysozyme, insulin, l-asparaginase, alteplase, bromelain, pegaspargase, and pancrelipase, which have been approved by the FDA, some enzymes like lysostaphin, endolysins, DNAse I, and alginate lyase are yet to be approved, as discussed in this review. All the enzymes discussed above are good alternatives to treat MDR pathogens, but the effectiveness of enzymes might be very less if they are used alone. To overcome this issue, different combinations of enzymes can be used to improve the efficacy of individual enzymes, but the individual enzyme selectively targets only specific sites.

To address the problems of low enzyme stability, high cost due to limited availability, difficulty to target specific areas, and inadequate delivery mechanisms, more research is required. Moreover, many enzymes are yet to be explored that can be used for treating various diseases and metabolic deficiencies. Even though this review mainly focuses on enzyme-based therapies, certain non-enzymatic inhibitors such as MurA inhibitors, DNA gyrase inhibitors, tazobactam, and clavulanic acid have also proven to be potential in addressing drug-resistant pathogens, which can be investigated for further study.

Enzyme-based therapeutics are emerging as a powerful strategy against MDR pathogens, offering precision killing, reduced likelihood of resistance development, and the ability to disrupt resilient biofilms. Unlike conventional antibiotics, these biologics can be engineered for specificity and synergy, complementing existing antimicrobial regimens. Recent progress in lysins, biofilm-degrading enzymes, and engineered catalytic proteins, coupled with advances in nanotechnology and synthetic biology, has brought enzyme therapy closer to clinical translation. Despite these promising developments, significant hurdles remain. Efficient delivery to infection sites, particularly in systemic and biofilm-associated infections, requires innovative vectors and smart release systems. Immunogenicity continues to pose a challenge, as foreign proteins may elicit neutralizing immune responses or adverse effects. Large-scale manufacturing and stabilization of enzymes at pharmaceutical grade remain costly and technically complex. Furthermore, regulatory pathways for enzyme therapeutics are still evolving, requiring robust efficacy metrics, long-term safety data, and harmonized global guidelines. Future directions in the field will probably focus on advanced protein engineering, multifunctional enzyme platforms, and integration with host-directed therapies to enhance immune modulation. With coordinated interdisciplinary and global efforts, enzyme-based antimicrobials could become a cornerstone of sustainable infectious disease management in the post-antibiotic era.

## Conflicts of interest

The authors declare that they have no known competing financial interests or personal relationships that could have appeared to influence the work reported in this paper.

## Abbreviations

AMPS: Antimicrobial peptides
CAZy: Carbohydrate-active enzymes
MDR: Multi-drug resistance
MPDA NPs: Mesoporous polydopamine nanoparticles
PDT: Photodynamic therapy
AOS: Alginate oligosaccharide
BMP-2: Bone morphogenetic protein-2
BPR: Biofilm percentage reduction
CDT: Chemo dynamic therapy
CF: Cystic fibrosis
COPD: Chronic obstructive pulmonary disease
E-DNA: Extracellular DNA
EPS: Exopolysaccharide
FDA: Food and drug administration
HRP: Horseradish peroxidase
LPS: Lipopolysaccharide
MBL: Metallo-β-lactamases
MIC: Minimum inhibitory concentration
MRSA: Methicillin-resistant *Staphylococcus aureus*
NETs: Neutrophil extracellular traps
PBPs: Penicillin-binding proteins
PIA: Polysaccharide intracellular adhesin
PLGA: Polylactic-*co*-glycolic acid
PLLs: Phosphotriesterase-like lactonases
PNAG: Poly-β-(1 → 6)-*N*-acetylglucosamine
POD: Peroxidase
PONS: Paraoxonases
PPH: Parathion protein hydrolase
PS: Photosensitizer
QQ: Quorum quenching
QS: Quorum sensing
ROS: Reactive oxygen species
SNPs: Single-nucleotide polymorphism
SOD: Superoxide dismutase
URE: Upstream regulatory element
VAP: Ventilator-associated pneumonia
WSPs: Water-soluble polysaccharides

## Data Availability

No primary research results, software or code have been included and no new data were generated or analysed as part of this review.
